# A narrow developmental window defines PKN2’s essential role in ventricular chamber morphogenesis

**DOI:** 10.1038/s42003-026-10287-9

**Published:** 2026-05-16

**Authors:** Julijus Bogomolovas, Zengming Zhang, Christa Trexler, Alex Campos, Jennifer Veevers, Zeyu Chen, Ju Chen

**Affiliations:** 1 Department of Medicine, UCSD, La Jolla, CA USA; 2 Proteomics Facility Core, Sanford Burnham Prebys Medical Discovery Institute, La Jolla, CA USA

**Keywords:** Musculoskeletal development, Development

## Abstract

Protein kinase N2 (PKN2) is essential for embryonic heart development, but the precise timing and cellular requirement in cardiomyocytes are not well understood. Here we show that, in a constitutive cardiomyocyte-specific knockout (cKO) of PKN2, we observe partially penetrant early postnatal lethality, non-progressive systolic dysfunction in survivors, and a distinctive “coin-pouch” ventricular geometry resembling the embryonic shape, linking the phenotype to a developmental origin rather than ongoing pathology. By tracking heart morphogenesis, we find that mutant and control hearts are still grossly indistinguishable at E10.5, defining a stage prior to overt morphological divergence. Integrative RNA-seq, proteomics, and phosphoproteomics at E10.5 reveal transcriptional and proteomic signatures consistent with induction of actin cytoskeleton and motility-associated programs alongside repression of chromosome-condensation and mitotic modules in PKN2 cKO hearts. These molecular changes coincide with reduced cardiomyocyte proliferation at E10.5–E11.5 and early divergence in ventricular-shape trajectories. Quantitative light-sheet microscopy–based morphometrics of inducible cardiomyocyte-specific PKN2 knockout hearts pinpoints the critical dependence window: tamoxifen administration at E7.5 reproduces the ventricular geometry characteristic of constitutive PKN2 cKO hearts, whereas administration at E10.5 spares gross morphology. Together, these data show that cardiomyocytes depend on PKN2 during an early proliferative phase, characterized by cytoskeletal remodeling and motility-associated programs, that contributes to ventricular wall formation, after which PKN2 becomes dispensable for gross ventricular chamber morphogenesis. This temporally restricted requirement reconciles previous observations of PKN2’s essential role in mesodermal and epithelial morphogenesis, suggesting a shared function in coordinating cytoskeletal remodeling with tissue expansion during organogenesis.

## Abstract (web summary)

Multi-omic and quantitative morphometric analyses define a narrow early developmental window during which cardiomyocytes require PKN2 for ventricular chamber morphogenesis in the mouse heart.

## Introduction

Heart development is a tightly coordinated process in which cardiomyocytes proliferate, differentiate, and migrate to assemble the multi-chambered embryonic heart. Precise regulation of these cellular events is essential for proper cardiac morphogenesis. Central to this regulation are intracellular signaling networks involving protein kinases, which orchestrate cardiomyocyte behavior during development. Among these, the protein kinase N (PKN) family has emerged as a key regulator in cardiovascular biology^1^. Notably, PKN2 plays a critical, non-redundant role in development. Global deletion of *Pkn2* in mice causes mid-gestational embryonic lethality, due to a failure of mesodermal expansion and cardiac development^2^. Consistent with its fundamental role, human genetic studies have linked variation at the PKN2 locus to increased cardiac disease risk, including myocardial infarction and ventricular tachyarrhythmias^3^. Despite this importance, the specific developmental processes governed by PKN2 in the embryonic heart have yet to be fully elucidated.

Initial insights into PKN2’s function during cardiogenesis came from conditional knockout models. Deleting *Pkn2* using the SM22α-Cre driver—active in cardiomyocytes but also in smooth muscle and other mesodermal lineages—resulted in partially penetrant embryonic lethality and cardiac abnormalities in mice^4,5^. Surviving SM22α-Cre; *Pkn2*^fl/fl^ neonates exhibited heart defects, indicating an essential developmental role for PKN2. However, because SM22α is expressed in multiple cell types, it was still unclear whether PKN2’s critical function was cardiomyocyte-intrinsic.

Subsequent studies employed a cardiomyocyte-specific Cre line (*XMLC2*-Cre) to target *Pkn2* deletion more precisely. This approach revealed a much more severe phenotype: cardiomyocyte-restricted PKN2 loss caused fully penetrant embryonic or perinatal lethality, with embryos displaying striking structural cardiac defects, such as numerous ventricular diverticula, large ventricular septal defects, and an exceedingly thin compact myocardium^5^. These findings established that PKN2 is indispensably required within cardiomyocytes for normal heart chamber formation. Nonetheless, the cellular mechanism underlying these malformations remained unclear. In particular, it was uncertain whether PKN2-deficient hearts failed due to impaired cardiomyocyte proliferation, altered differentiation, aberrant cell migration, or other developmental disruptions. Intriguingly, PKN2’s necessity appears to be developmentally stage-specific. Whereas its absence in embryonic cardiomyocytes is catastrophic, the combined knockout of *Pkn1* and *Pkn2* in adult cardiomyocytes has a minimal baseline effect and can even be protective under stress by blunting heart failure progression in pressure overload models^6^. This stark contrast highlights a crucial unanswered question – what does PKN2 do during early heart development that becomes dispensable in the adult heart?

To answer this question, we identified embryonic day 10.5 (E10.5) as the latest developmental point when hearts are grossly indistinguishable between *Pkn2* cKO and control animals.

Therefore, we selected E10.5 as a critical stage to examine molecular changes precipitating the phenotype, allowing us to capture primary gene and protein expression alterations before secondary effects and heart failure confound the picture. Transcriptomic (RNA-seq) and (phosphor)proteomic analyses of E10.5 *Pkn2*-knockout hearts revealed an enrichment of gene sets related to the actin cytoskeleton and cell migration. These signatures are consistent with a model in which loss of PKN2 perturbs coordinated cytoskeletal and cell-cycle gene programs in cardiomyocytes, contributing to the structural defects in the developing heart.

To directly test the hypothesis that PKN2 is specifically required during the early morphogenetic window characterized by cytoskeletal remodeling and proliferation^7^, we employed an inducible, cardiomyocyte-restricted *Pkn2* knockout strategy. Using a tamoxifen-inducible Tnnt2-MerCreMer driver^8^, we timed *Pkn2* deletion at distinct embryonic stages. Strikingly, when PKN2 deletion was triggered early, following tamoxifen administration at E7.5 during the initial stages of ventricular wall formation, embryos developed severe cardiac abnormalities closely resembling those observed in the constitutive knockout. In contrast, tamoxifen administration at E10.5, a stage by which cardiomyocyte clonal expansion and motility-associated transcriptional programs are shown to decline^7^ resulted in hearts with largely preserved gross ventricular chamber geometry. These findings pinpoint a narrow developmental window in which PKN2 is critically required. During this early window, PKN2 likely contributes to cytoskeletal organization and motility-associated programs in cardiomyocytes, thereby ensuring proper compaction and chamber morphogenesis. Once this early morphogenetic phase concludes, PKN2 becomes largely dispensable for gross ventricular chamber morphogenesis.

## Results

### Loss of cardiomyocyte PKN2 leads to partial early postnatal lethality with profound structural and functional heart defects

**Fig. 1:**
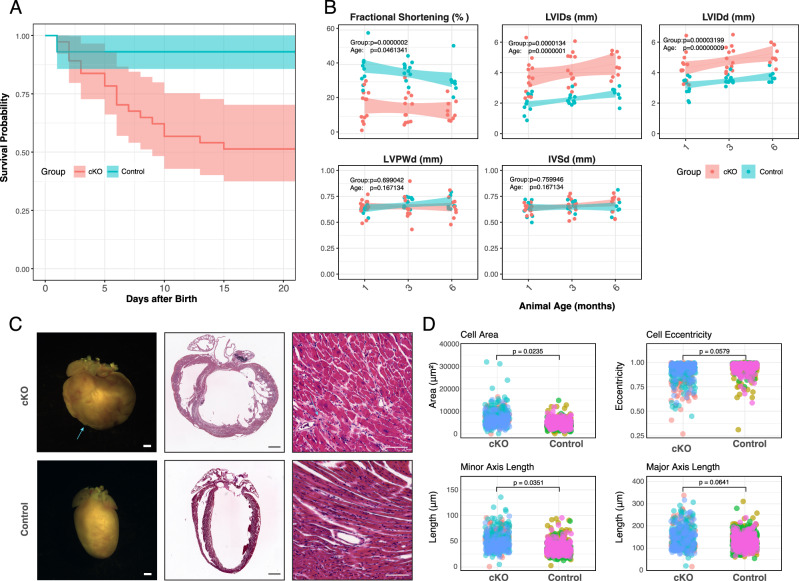
Cardiomyocyte PKN2 is required for early postnatal survival, normal ventricular geometry and cardiomyocyte growth. **A** Neonatal survival. Kaplan–Meier analysis shows marked perinatal mortality in the *Pkn2* cardiomyocyte-specific knockout (cKO), with survival plateauing by postnatal day 20 (cKO, *n* = 37; controls, *n* = 43; biologically independent; two‑sided log‑rank test *p* = 0.00004305, ribbon represents 95% CI). **B** Longitudinal echocardiography. Surviving cKO animals exhibit non‑progressive systolic dysfunction and chamber dilation without a significant change in ventricular wall thickness. Shown are fractional shortening (FS), LV internal diameter at systole (LVIDs) and diastole (LVIDd), LV posterior wall thickness at diastole (LVPWd), and interventricular septal thickness at diastole (IVSd) measured across ages (months). Ribbons depict robust linear fits with 95% CIs and are used for visualization only; individual points are animals at each time point (*n* per time point: 9–13 cKO and 7–11 controls). *P* values for main effects are printed on each panel. **C** Gross and histological pathology at P30. cKO hearts display a characteristic rounded “coin‑pouch” external morphology and abnormal internal architecture, including ventricular diverticula and disorganized myocardium with cardiomyocyte disarray. Representative whole mount images (left arrow indicates diverticulum), whole‑section H&E (middle), and high‑magnification myocardial views (right, arrow indicates area of clustered myocyte disarray) are shown; scale bars as indicated on panels (1 mm for whole mount and low magnification H&E; 0.1 mm for high magnification H&E). **D** Cardiomyocyte morphometry. Quantification of isolated LV cardiomyocytes from P30 animals (*n* = 3 biologically independent hearts per group) shows increased cell area and minor axis length (cell thickness) in cKO, with no significant change in eccentricity (roundness) or major axis length (cell length). Points are individual cells, colored by biological replicate; *P* values (top of each panel) are from mixed‑effects modeling with mouse as a random effect.

To determine whether *Pkn2* is expressed during the developmental window relevant to ventricular morphogenesis, we reanalyzed a published embryonic mouse heart single-cell RNA-seq dataset (GSE76118)^9^. *Pkn2* transcripts were detected across multiple anatomical regions of the developing heart and across early developmental stages, indicating that *Pkn2* is broadly expressed during early cardiac morphogenesis (Supplementary Fig. S1). To examine the role of PKN2 in cardiomyocytes, we generated a cardiomyocyte-specific knockout (cKO) mouse model using a floxed *Pkn2* allele and the *XMLC2*-Cre line, which drives highly specific recombination in the cardiomyocytes from early development^10^. Animals were born at the expected Mendelian ratio but began dying shortly after birth, with a plateau by approximately postnatal day 20 (P20), resulting in about 50% survival of *Pkn2* cKO animals beyond this period (Fig. 1A). Longitudinal echocardiography of survivors revealed a persistent cardiac dysfunction (Fig. 1B). Knockout animals showed persistent reduced fractional shortening and ventricular dilation compared with control littermates. Notably, ventricular wall thickness (IVSd, LVPWd) was unchanged. Mixed-effects modeling of serial measurements identified significant effects of genotype and age but no genotype-by-age interaction. This means that systolic dysfunction and chamber dilation in *Pkn2* cKO hearts were already present at the earliest measured time point and progressed in parallel with normal postnatal growth, indicating a stable, non-progressive phenotype. The significant age effect reflected normal growth-related scaling of cardiac dimensions in developing mice. Gross examination of *Pkn2* cKO hearts at P30 revealed striking structural abnormalities (Fig. 1C). The hearts appeared enlarged and severely misshapen, adopting a characteristic “coin-pouch” morphology. Histological analysis confirmed a disorganized myocardial architecture: cKO ventricles exhibited cardiomyocyte disarray and poor compaction, in contrast to the orderly alignment seen in controls. To further assess cardiomyocyte geometry, we performed morphometric analysis on isolated ventricular myocytes (Fig. 1D and Supplementary Fig. 5). *Pkn2* cKO cardiomyocytes displayed a significant increase in overall area, consistent with hypertrophy, and an increase in cell thickness (minor axis length). However, cell length (major axis) was not significantly altered relative to controls. In classic dilated cardiomyopathy (DCM), by contrast, cardiomyocytes typically undergo eccentric hypertrophy characterized by elongation (addition of sarcomeres in series) with insufficient thickening—cellular remodeling that contributes to chamber dilation and wall thinning in failing hearts^7^. Thus, while cardiomyocyte PKN2 deletion induces a dilated, hypocontractile phenotype, the stable course and absence of typical elongation-driven remodeling indicate that it does not fully recapitulate a classical DCM progression.

### Multi-omic analysis of *Pkn2* cKO hearts at E10.5, ahead of gross morphological changes, reveals early induction of actin cytoskeletal remodeling and repression of proliferative modules

**Fig. 2:**
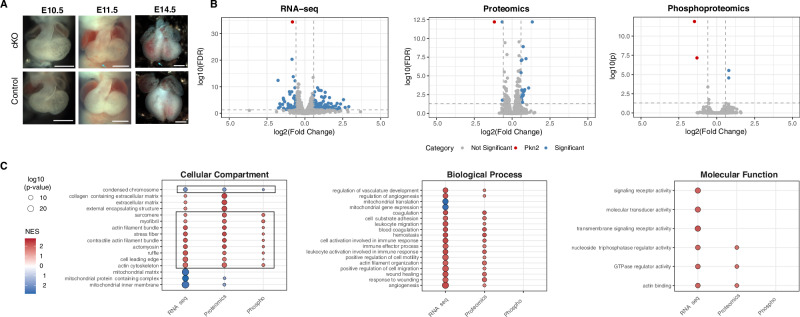
Early morphological divergence and multi-omic gene-set shifts in PKN2 cKO hearts. **A** Whole mount morphology of control and *Pkn2* cKO hearts at E10.5, E11.5 and E14.5. At E10.5, hearts are macroscopically indistinguishable. By E11.5, the cKO ventricular surface appears rougher and less regular (arrows indicate prominent irregularities of ventricular walls), despite overall chamber outlines remaining similar. At E14.5, control hearts narrow and elongate along the base–apex axis with progressive loss of interventricular sulcus, whereas cKO hearts remain broader and more bulbous, retaining an early-embryonic–like contour (scale bar 0.5 mm). **B** Volcano plots of differential features (RNA, protein, phospho-site) comparing cKO vs. control at E10.5. *Pkn2* is appropriately depleted (red dots); few additional features surpass |log₂FC| > log₂(1.5) and FDR (or *p*) < 0.05 (blue dots), while gray points indicate non-significant features. **C** Cross-omic gene set enrichment summary across RNA, proteome, and phosphoproteome (phospho protein-level scores aggregated from site-level z-scores via Stouffer’s method). Each dot’s size encodes –log₁₀(*p*) and fill encodes normalized enrichment score (NES; red = enriched in cKO, blue = depleted). Boxes denote gene sets directionally concordant across all three modalities.

Analysis of postnatal PKN2 cKO animals revealed severe, but largely compensated, non-progressive cardiac dysfunction. Therefore, we speculated that this phenotype is rooted in a defect in cardiac development. Taking into consideration that loss of PKN2 causes prominent morphological abnormalities, we sought to determine when the PKN2 loss phenotype first becomes apparent. We examined whole-mount hearts at E10.5, E11.5, and E14.5. At E10.5, cKO and control hearts were indistinguishable at the gross level (Fig. 2A). By E11.5, overall chamber geometry remained similar, but the cKO ventricular surface appeared rougher and less regular than in control hearts. By E14.5, control hearts displayed the expected narrowing and elongation along the base–apex axis with progressive fusion of the early embryonic bifid apex, whereas cKO hearts remained broader and more bulbous, retaining an early embryonic-like external contour. Thus, we selected E10.5 as a stage prior to overt morphological divergence for molecular profiling, allowing us to focus on early molecular consequences of cardiomyocyte-specific loss of PKN2 in the developing heart. We performed RNA sequencing (RNA-seq; 4 cKO vs. 4 controls), whole proteome mass spectrometry (five pooled “metasamples” per genotype, each derived from ~20 hearts; total ~102 hearts/genotype), and phosphoproteomics analysis. Per-feature differential testing yielded the expected depletion of *Pkn2* across modalities but otherwise few large-effect hits at |log₂FC| > log₂(1.5) and FDR (or *q*) < 0.05 (Fig. 2B), indicating that molecular changes at this stage are relatively subtle at the level of individual genes, proteins or phosphosites.

Because PKN2 is a serine/threonine kinase, one immediate molecular consequence of its loss would be altered phosphorylation of its substrate proteins. As a first probe of this expectation, we examined phosphopeptides corresponding to reported PKN2 substrates detected in our phosphoproteomic dataset (Supplementary Fig. 3 and Supplementary Data 1). While several substrate-associated phosphosites were identified, their changes were generally modest and did not follow a uniform directional pattern, with some sites increased and others decreased in cKO hearts. This substrate-focused analysis, therefore, served as an internal probe of proximal signaling consequences of PKN2 loss and suggested that early phosphorylation changes are subtle and distributed rather than dominated by a single substrate event.

We therefore adopted a pathway-level approach and performed gene set enrichment analysis (GSEA) on ranked RNA-seq, proteomic and phosphoproteomic feature lists to identify coordinated biological programs associated with early PKN2 deficiency in cardiomyocytes. Across all three modalities, we detected enrichment of actin cytoskeleton-related gene sets in cKO hearts (*sarcomere*, *myofibril*, *actin filament bundle*, *contractile actin filament bundle*, *actomyosin*, *stress fiber*, *ruffle*, *cell leading edge* and *actin cytoskeleton*) (black-marked rows in Fig. 2C). Additional related terms showed consistent directionality in two of the three assays, including *actin binding* and *regulation of cell motility*. In contrast, *chromosome condensation* was the only gene set consistently down-enriched across all three modalities. Taken together, despite modest per-feature differences at E10.5, cross-modality enrichment analysis identifies coordinated cytoskeletal and chromosome-associated programs linked to early PKN2 loss, providing molecular context for the developmental phenotype that emerges at later stages.

### Early loss of PKN2 in cardiomyocytes affects ventricular geometry and the proliferation of developing cardiomyocytes

Our integrated multi-omics analysis of *Pkn2* cKO embryonic hearts identified two major themes: an upregulation of actin/cytoskeletal gene sets and a downregulation of condensed chromosome gene sets at E10.5. It has been shown that there is a short developmental window in mouse hearts (before ~E12.5) when cytoskeleton-dependent motility and proliferation contribute robustly to ventricular wall growth, after which cardiomyocytes become more growth-arrested and less migratory^7^.

**Fig. 3:**
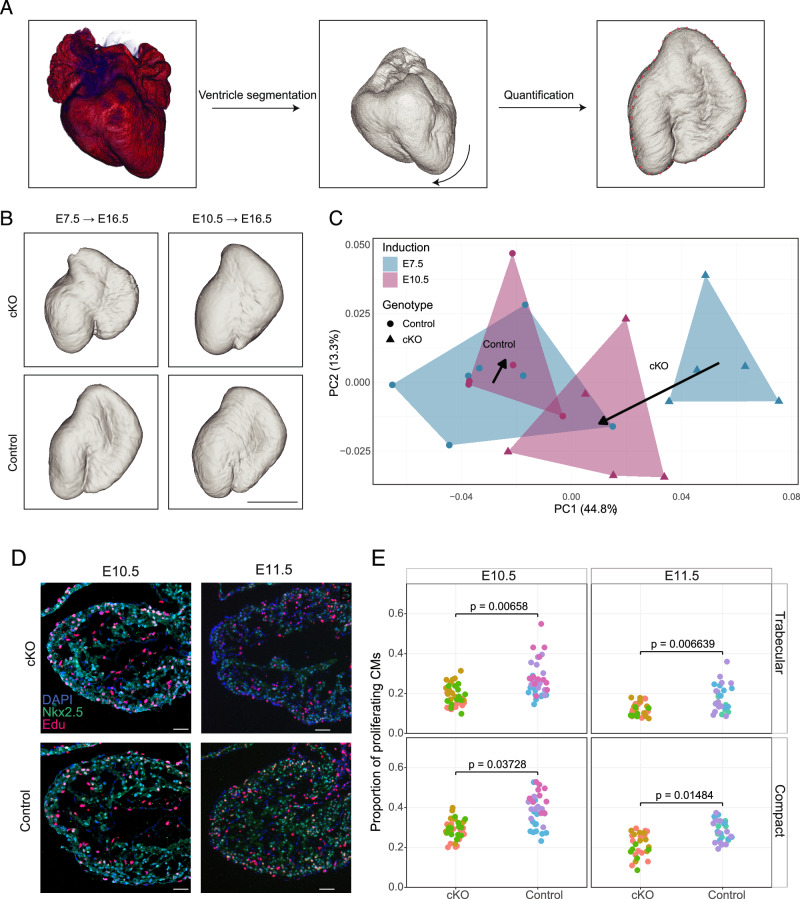
PKN2 is required for cardiomyocyte proliferation and proper ventricular morphogenesis during a narrow developmental window. **A** Workflow for Three-Dimensional Ventricular Shape Quantification. Cleared embryonic hearts were imaged by light-sheet microscopy, ventricular regions were segmented to generate surface reconstructions, and sliding semilandmarks were placed along a homologous contour of the dorsal ventricular surface to enable geometric morphometric analysis. **B** Representative reconstructions of E16.5 ventricles from control and *Pkn2* cKO hearts following early (E7.5) or late (E10.5) Tnnt2^MerCreMer^ induction. Early deletion (E7.5) produces broadened and abnormal ventricular geometry in *Pkn2* cKO hearts, whereas deletion at E10.5 yields ventricles largely comparable to controls. Scale bar, 500 µm. **C** Principal component analysis (PCA) of ventricular shapes in Procrustes morphospace. Each point represents an individual heart (*n* = 5/7 cKO/control at E7.5 and 5/5 at E10.5 induction; biologically independent embryos). Polygons outline the distribution of each experimental group, and arrows connect centroid positions for control and cKO groups within each induction condition, indicating the direction and magnitude of genotype-specific shape trajectories. Early (E7.5) cKO hearts diverge markedly from controls, whereas E10.5-induced cKO hearts cluster closer to control shapes. **D** Representative immunofluorescence images of E10.5 and E11.5 heart sections co-stained for EdU (red) and the cardiomyocyte nuclear marker NKX2.5 (green); nuclei counterstained with DAPI (blue). *Pkn2* cKO hearts display fewer EdU⁺ cardiomyocyte nuclei compared to controls. Scale bars, 50 µm. **E** Quantification of cardiomyocyte proliferation. Proportion of EdU⁺/NKX2.5⁺ nuclei among total NKX2.5⁺ cardiomyocyte nuclei in trabecular and compact myocardium at E10.5 and E11.5. Each dot represents an individual microscopic field (color-coded by animal; *n* = 3 biologically independent animals per genotype per stage). *P* values were obtained from generalized linear mixed-effects modeling with genotype and stage as fixed effects and animal as a random intercept. *Pkn2* cKO hearts show significantly reduced cardiomyocyte proliferation at both developmental stages and in both myocardial compartments.

Based on this background, we hypothesized that loss of PKN2 during the early, active-morphogenesis window would distort heart development, whereas deleting PKN2 at slightly later stages (when heart growth relies less on cytoskeletal remodeling and motility-associated processes) would have milder effects on cardiac shape. To test this, we used a tamoxifen-inducible Tnnt2^MerCreMer^ driver^8^ to achieve cardiomyocyte-specific PKN2 knockout at defined time points and examined the resulting ventricular morphology. We induced recombination at E7.5 (early) or E10.5 (late), and harvested hearts at E16.5 for morphological analysis. Animals carried the *Rosa26*^mT/mG^ reporter allele to allow direct visualization of recombined cardiomyocytes. Hearts were optically cleared and imaged by light-sheet microscopy. Ventricular surface reconstructions used for morphometric analysis were segmented from the membrane-targeted GFP signal marking recombined cardiomyocytes (Fig. 3A). To verify that recombination was not restricted to superficial myocardial layers, we examined optical sections from cleared hearts. GFP labeling was widespread throughout the ventricular myocardium following both E7.5 and E10.5 induction, supporting efficient recombination across the ventricular wall (Supplementary Fig. 2). Deleting PKN2 in early cardiogenesis (E7.5 induction) led to striking ventricular shape abnormalities by E16.5, with reconstructed ventricles appearing broader and less elongated than controls, whereas deletion at E10.5 produced ventricles that were largely similar in shape to control hearts (Fig. 3B). To quantitatively compare ventricular shape across samples, we next performed geometric morphometric analysis using sliding semilandmarks placed along a homologous contour of the dorsal ventricular surface (Fig. 3A). This contour traces the major ventricular outline, spanning the bifid ventricular apex region and extending toward the outflow tract, enabling consistent comparison of chamber geometry across specimens. We then used generalized procrustes analysis (GPA), followed by Shape ANOVA and phenotypic trajectory analysis to quantify morphological differences. Across all specimens, the first two principal components (PC1 and PC2) captured 44.8% and 13.3% of shape variance, respectively. Visualization of the ±2 SD shape extremes indicates that PC1 primarily reflects global ventricular contour changes, spanning elongated to broadened ventricular geometries (Supplementary Fig. 4). Consistent with this interpretation, ventricles from E7.5-induced *Pkn2* cKO hearts project toward the positive end of PC1, corresponding to the broadened contour visible in the reconstructed ventricular surfaces, whereas control and E10.5-induced cKO hearts cluster toward the negative side of the axis (Fig. 3B). Shape ANOVA revealed a highly significant main effect of genotype on ventricular shape (procrustes ANOVA: *F* = 9.29, *p* = 0.0001) and a significant induction × genotype interaction (*F* = 2.45, *p* = 0.0157), indicating that the impact of PKN2 loss on heart shape depends on when the knockout was induced. Consistent with this result, phenotypic trajectory analysis showed that the magnitude of shape change from control to cKO was much greater when PKN2 was deleted at E7.5 than when deleted at E10.5. The Procrustes distance between control and cKO shape means was 0.083 for early induction versus 0.046 for late induction, and this difference in path length (Δ = 0.037) was significant (*p* = 0.025). Moreover, the direction of shape change in morphospace differed between early and late knockouts (trajectory angle = 48.4°, *p* = 0.0044), indicating that the direction of ventricular shape change depends on the developmental timing of PKN2 deletion.

In summary, PKN2 deletion at E7.5 caused a larger and qualitatively different ventricular shape deviation compared to controls, whereas deletion at E10.5 resulted in only a minor deviation, with cKO hearts closely resembling control hearts.

Because our multi-omics analysis at E10.5 indicated downregulation of chromosome condensation gene sets in *Pkn2* cKO hearts, we next assessed cardiomyocyte proliferation using EdU (5-ethynyl-2′-deoxyuridine) incorporation. EdU labeling marks cells that have undergone DNA synthesis during the 2 h labeling window. By co-immunostaining for the cardiomyocyte nuclear marker NKX2.5, we quantified cardiomyocyte nuclei that incorporated EdU in heart sections. We focused on E10.5 and E11.5, the developmental stages profiled in our multi-omics datasets. In both stages, PKN2 cKO hearts showed a significant reduction in cardiomyocyte proliferation compared to controls (Fig. 3D, E). For example, at E10.5, the fraction of EdU⁺/NKX2.5⁺ cardiomyocyte nuclei in cKO hearts was ~80% of that observed in controls (*p* < 0.01). A similar reduction in proliferation was observed at E11.5. Regional quantification further showed that this proliferation defect was present in both the compact and trabecular myocardium, indicating that the reduction in cardiomyocyte proliferation is not restricted to a specific ventricular compartment. To exclude the possibility that the reduced EdU⁺/NKX2.5⁺ fraction reflected a change in cardiomyocyte abundance rather than proliferative rate, we quantified total NKX2.5⁺ nuclei relative to total DAPI⁺ nuclei across stages and regions. A mixed-effects count model showed no significant genotype effect on cardiomyocyte proportion (*χ*² = 2.68, d*f* = 1, *p* = 0.102) and no genotype interactions with stage or region (all *p* ≥ 0.169).

Taken together, these results demonstrate that PKN2 function is critically required during an early developmental window for normal cardiomyocyte proliferation and ventricular morphogenesis. Loss of PKN2 during this period is associated with reduced cardiomyocyte proliferation and significant, quantifiable ventricular shape abnormalities by late gestation. In contrast, deletion of PKN2 at later stages results in only minor deviations in ventricular chamber geometry, indicating that the impact of PKN2 loss on ventricular shape depends strongly on the developmental timing of gene inactivation.

## Discussion

Our multi-omics analysis at E10.5, the last stage before overt morphological divergence, provides molecular context for the phenotype. Across transcriptome, proteome, and phosphoproteome, we observed concordant up-enrichment of actin cytoskeleton and motility-associated gene sets, accompanied by down-enrichment of chromosome-condensation and cell-cycle programs. These molecular signatures correspond to the developmental transition identified by Sereti et al., during which, between E9.5 and E12.5, cardiomyocyte clonal expansion capacity and expression of motility-associated gene programs progressively decline^7^. In that context, loss of PKN2 during the brief proliferative and cytoskeletal remodeling phase likely perturbs cellular processes that contribute to chamber shaping, leading to ventricular shape distortion that persists once the proliferative window closes. Importantly, our study does not directly measure cardiomyocyte migration or division orientation in vivo. The evidence for altered motility-associated programs rests on pathway-level enrichment of cytoskeletal and motility-associated gene sets across three independent molecular modalities. What we do demonstrate directly is a significant reduction in cardiomyocyte proliferation (EdU/NKX2.5) during the critical E10.5 to E11.5 interval. Whether the cytoskeletal changes also reflect altered migratory or polarization behavior in PKN2-deficient cardiomyocytes remains an open question for future live-imaging studies.

This interpretation is further supported by PKN2’s established roles in other cell types. PKN2 is a Rho/Rac-responsive effector that regulates cytoskeletal organization, cell migration, and cell-cycle progression^1,4,11–13^. Global *Pkn2* knockout causes embryonic lethality with mesodermal expansion failure, and PKN2-deficient primary fibroblasts exhibit reduced proliferation and migration^2,4^. Thus, PKN2 likely performs related functions in developing cardiomyocytes, coordinating cytoskeletal dynamics and proliferation during a critical morphogenetic window, consistent with its broader cellular roles.

Our findings complement prior in vivo cardiac PKN2 studies. Marshall et al.^5^ used the same *XMLC2*-Cre driver to achieve cardiomyocyte-directed PKN2 deletion and reported frequent myocardial clefts/diverticula, ventricular septal defects, and abnormal heart shape, with almost no homozygous animals surviving postnatally^5^. The more penetrant embryonic and early postnatal lethality in their study likely reflects genetic background differences, as Marshall et al. maintained lines on C57BL/6J, whereas we used a C57BL/6N background. These substrains differ at functional alleles and exhibit distinct cardiovascular phenotypes and stress responses^14,15^. Such background effects can substantially modify the penetrance and expressivity of cardiac defects. Nonetheless, both studies found that loss of PKN2 in cardiomyocytes leads to severe structural heart defects arising from disrupted early morphogenesis, highlighting the essential developmental role of PKN2 across genetic contexts.

Notably, both studies employed the same floxed allele and Cre driver, isolating genetic background as the key variable influencing penetrance—a reminder that phenotypic outcomes in conditional knockout models are sensitive to factors beyond the target gene itself, whether genetic background, as observed here, or the design of the targeted allele, as recently demonstrated at the Atf4 locus^16^. Notably, the ventricular abnormalities observed in *Pkn2* cKO hearts, including ventricular diverticula/outpouchings, cardiomyocyte disarray, and impaired myocardial compaction, share features with congenital disorders of ventricular wall maturation, such as left ventricular noncompaction and congenital ventricular diverticulum^17–20^. Although no pathogenic PKN2 variants have been reported in these conditions to date, the phenotypic overlap suggests that PKN2-dependent signaling during early chamber morphogenesis may be relevant to the pathogenesis of human ventricular wall defects. More broadly, the cellular processes that PKN2 coordinates during this developmental window, namely cardiomyocyte proliferation and cytoskeletal remodeling, are also central to myocardial repair. Neonatal mammalian heart regeneration involves cardiomyocyte proliferation and ingrowth into the injury zone^21^, and zebrafish heart regeneration depends on cardiomyocyte migration toward the wound^22^. The fact that PKN2 is essential during the developmental window when these capacities are high, and dispensable once they decline, suggests that PKN2 activity may be a feature of the proliferation-competent cardiomyocyte state. Whether PKN2 could serve as a marker of regenerative potential, or whether its modulation could influence cardiomyocyte proliferative responses in therapeutic settings, remains to be explored.

Several limitations frame natural extensions of this work. Our cytoskeletal interpretation rests on cross-modality pathway-level enrichment rather than direct measurement of actin organization, polarity, or migration, which will require targeted live-imaging in future studies. The observed proliferation reduction we view as one component of a multifactorial defect that compounds with cytoskeletal program changes within the narrow morphogenetic window. Our two induction time points (E7.5, E10.5) were chosen to bracket this window; finer temporal resolution will refine its boundaries.

Collectively, our work refines the role of PKN2 during cardiogenesis by identifying a critical developmental window around E10.5 beyond which PKN2 becomes largely dispensable for gross ventricular chamber morphogenesis, demonstrating causally via inducible deletion that only early cardiomyocytes require PKN2, and linking multi-omics signatures to quantified ventricular geometry and reduced proliferation within the established developmental window of cardiomyocyte clonal expansion and cytoskeletal remodeling. Our data indicate that PKN2 in cardiomyocytes performs functions analogous to those described in other cell types, coordinating cytoskeletal dynamics and cell-cycle progression during a finite period of active tissue remodeling. Once this early morphogenetic phase wanes, PKN2 is no longer required for gross ventricular chamber morphogenesis.

## Methods

### Murine models

Mice carrying a floxed *Pkn2* allele (exon 8 flanked by LoxP sites; EMMA ID: 09415) were purchased from European Mouse Mutant Archive and maintained in a specific pathogen-free facility. Mice were housed 2–5 per cage with ad libitum access to Teklad Diet 2020X and water via an automated system, under environmental controls maintaining 21–23 °C, 45–55% relative humidity, and a 12:12 h light:dark cycle (lights on/off at 6 AM/6 PM). We have complied with all relevant ethical regulations for animal use. All procedures were approved by our Institutional Animal Care and Use Committee (IACUC) under protocol S01049, in compliance with NIH and institutional guidelines. Animals were monitored routinely according to IACUC-approved welfare criteria, and predefined humane endpoints were applied as required by the protocol (including weight loss, severe lethargy, inability to feed and respiratory distress). Euthanasia for tissue collection was performed using IACUC-approved methods, with postnatal animals euthanized by isoflurane anesthesia followed by cervical dislocation and confirmation of death by cessation of respiration and heartbeat. Embryos were collected following maternal euthanasia under the same protocol. To remove the selection cassette from the original Pkn2tm1a(KOMP)Wtsi allele, mice were first crossed with a ubiquitous FLP deleter line to excise the FRT-flanked LacZ/Neo cassette, and subsequently the FLP transgene was bred out. For constitutive deletion of *Pkn2* in cardiomyocytes, we preserved a homozygous floxed line (*Pkn2*^fl/fl^) and crossed these mice with heterozygous *XMLC2*-Cre animals; experimental mice were *Pkn2*^fl/fl^; Cre+, and littermate Cre-negative flox/flox mice served as controls. For inducible deletion experiments, we maintained a *Pkn2*^fl/fl^; *Rosa26*^mT/mG/ mT/mG^ stock and crossed them with Tnnt2^MerCreMer+^; *Pkn2*^fl/+^ mice so that all progeny inherited the reporter allele (*Rosa26*^mT/mG/+^), and *Pkn2*^fl/fl^; Tnnt2^MerCreMer+^ mice were designated as experimental, while *Pkn2*^fl/+^; Tnnt2^MerCreMer+^ mice served as controls. Recombination in utero was induced by voluntary administration, with pregnant dams receiving 1 mg tamoxifen dissolved in corn oil and delivered in a sweetened milk formulation by micropipette feeding at the specified time points^23^. All lines were maintained on a C57BL/6N genetic background. Both male and female mice were used for experiments, as no significant sex-dependent differences in cardiac function were detected by echocardiography in PKN2 cKO animals. For timed pregnancies, embryonic day 0.5 (E0.5) was defined as vaginal plug detection in the morning after mating. Group allocation was genotype-defined; thus, no additional randomization was used. Unless otherwise specified, the experimental unit was one animal (mouse or embryo).

### Echocardiography

Transthoracic echocardiography was performed as previously described in our laboratory’s publications^24,25^. Briefly, mice were anesthetized by inhalation of 5% isoflurane for induction (≈15 s) and maintained at 0.5–1% isoflurane in oxygen during imaging. Chest hair was removed with depilatory cream to optimize acoustic coupling. During the exam, mice were positioned supine on a heated platform, and heart rate was continuously monitored. Echocardiography was conducted using a FUJIFILM VisualSonics Vevo 2100 high-resolution ultrasound system equipped with a 32–55 MHz linear transducer. Two-dimensional B-mode and M-mode images were obtained from parasternal long- and short-axis views. Left-ventricular internal dimensions at end-diastole (LVIDd) and end-systole (LVIDs), and posterior wall thickness in diastole (LVPWd) were measured from M-mode tracings at the mid-papillary level. Fractional shortening (FS) was calculated as an index of systolic function using FS = [(LVIDd − LVIDs)/LVIDd] × 100%. Heart rates during image acquisition ranged from approximately 500 to 660 bpm (mean 563 bpm), indicating that anesthesia levels maintained physiological cardiac activity without bradycardic depression. All echocardiographic measurements were performed by an operator blinded to mouse genotype, and the investigator was masked to group assignment during image acquisition and analysis.

### Integrated analysis of transcriptome, proteome and phosphoproteome

Tissue from 102 cKO and 102 control hearts was pooled into five groups per genotype and lysed in 8 M urea, 50 mM ammonium bicarbonate. Lysates were clarified, normalized for protein concentration, TMT 10‑plex labeled, and analyzed by LC–MS/MS at the Proteomics Facility Core, Sanford Burnham Prebys Medical Discovery Institute, using the core’s standard sample‑preparation, acquisition, and processing workflows. Reporter ion intensities were log₂-transformed and analyzed in R using DEP2: variance-stabilizing normalization across TMT channels, missing values imputed by random-forest imputation^26^, and differential abundance between cKO and control tested using limma modeling with Strimmer’s *q*-value FDR correction; significance was defined as adjusted *p* < 0.05 and |log₂-fold-change| ≥ 1. Meanwhile, total RNA from ventricular tissue of four cKO and four littermate control samples was extracted with TRIzol (Invitrogen), cDNA libraries built using the TruSeq RNA Library Prep Kit v2 (Illumina), and sequenced on a NovaSeq X Plus instrument (150 bp paired-end). Raw FASTQs were processed with the nf-core/rnaseq workflow (v3.14.0) using the GRCm39 mouse reference genome and annotation to generate QC metrics, alignments, and a gene-expression count matrix, which was imported into R and analyzed with DESeq2 (v1.44.0)^27^ using design ~ condition (control vs. cKO) after filtering to retain genes with total counts ≥ number of samples; genes with |log₂FC| ≥ log₂(1.5) and FDR ≤ 0.05 were called significant.

For multi-omics integration, we used three ranked variables: RNA log₂ fold-change from the RNA-seq assay, protein log₂ fold-change from the proteomics assay, and phospho protein-level z-scores obtained by aggregating site-level z-scores via Stouffer’s method^28^, to perform GSEA for the Gene Ontology categories (GO/CC/MF/BP) using the R package clusterProfiler^29^. We then selected the top 25 GO terms within each ontology by ranking terms according to combined −log₁₀(*p*-value) within modality, and visualized whether these terms appeared in one, two, or all three modalities by generating volcano plots for each modality and a pattern-grouped dot plot where point size corresponds to −log_10_(raw *p*) and fill color indicates normalized enrichment score (NES).

### Ventricle shape analysis

Embryonic hearts were dissected, fixed overnight in 4% paraformaldehyde at 4 °C, and subsequently cleared using CUBIC‑L reagent^30^. After clearing, hearts were embedded in low-melting-point agarose cylinders supplemented with 0.5 µm fluorescent beads for fiduciary registration and equilibrated in refractive-index-matching solution (RIMS)^31^. They were imaged on a Zeiss Light Sheet Z.1 microscope using multi-angle acquisition to capture high-resolution volumetric data. Raw multiview datasets were then registered, fused, and deconvolved using BigStitcher^32^. Following image processing, ventricular regions were sparsely annotated in 3D Slicer^33^ and used as training data for a deep learning-based segmentation implemented in nnU-Net^34^. The resulting binary ventricle masks were converted to 3D surface models. To quantify ventricular shape, sliding semilandmarks were placed along a homologous contour of the dorsal ventricular surface spanning the bifid ventricular apex and extending toward the outflow tract, providing a reproducible anatomical outline across specimens. A total of 50 semilandmarks were defined on a template ventricle and transferred across specimens using the ALPACA pipeline^35^. Semi-landmarks were allowed to slide along tangents to minimize bending energy and improve geometrical correspondence across samples. Landmark configurations were subjected to a GPA to remove variation due to size, orientation and translation. Subsequently, shape variation and differences among groups were assessed using procrustes analysis of variance in the R package geomorph^36,37^ and residual-randomization permutation procedures (RRPP) in the R package RRPP^38,39^. Principal component analysis of the procrustes-aligned coordinates was used to visualize major axes of shape variation, and phenotypic trajectory analysis compared the magnitude and orientation of shape change between genotypes across induction timepoints.

### Histological analyses

Histological analyses were performed as described previously^40,41^. For proliferation assays, pregnant dams received an intraperitoneal injection of 30 mg/kg EdU (5-ethynyl-2′-deoxyuridine) 2 h prior to embryo harvest. Embryos were fixed in PFA, embedded in OCT, and cryosectioned for proliferation assessment. EdU incorporation was detected using the Click-iT™ EdU kit according to the manufacturer’s instructions. Cardiomyocyte nuclei were identified by immunostaining for NKX2-5 (Santa Cruz Biotech, SC8697, RRID:AB_650280), and nuclei were counterstained with DAPI. EdU⁺ nuclei within the NKX2-5⁺ cardiomyocyte population were quantified and normalized to the total number of NKX2-5⁺ nuclei per section, following the count-regression framework^42^. For adult heart morphology, adult hearts were fixed in PFA, paraffin-embedded, sectioned and stained with hematoxylin and eosin using standard protocols. Images were acquired using either an Olympus SLIDEVIEW VS200 slide scanner or an ECHO Revolution microscope.

### Cardiomyocyte isolation and morphometric analysis

Cardiac myocytes were isolated via retrograde Langendorff perfusion, as previously described^25^. Immediately upon isolation, cells were fixed in 4% paraformaldehyde and mounted on glass microscopy slides. Nuclei were counterstained with DAPI, while cardiomyocyte bodies were visualized via their intrinsic green autofluorescence. Imaging was performed on a Leica SP8 confocal microscope. Image stacks were then processed using a custom workflow. First, the *cyto3* model within Cellpose^43,44^ was fine-tuned through an iterative process applied to our image set, with the explicit aim of minimizing over-segmentation of cardiomyocytes. The resulting segmentation masks and their corresponding raw images were imported into CellProfiler^45^ for feature extraction. A range of morphometric measurements were computed in CellProfiler, and data were exported to CellProfiler Analyst^46^ for classification. In this step, hyper-contracted or otherwise morphologically abnormal cardiomyocytes were excluded through a trained gating procedure. Masks corresponding to morphologically valid cardiomyocytes were retained for quantitative analysis. All reported morphometric parameters derive from the accepted cardiomyocyte masks as output by CellProfiler.

### Statistics and reproducibility

All analyses were performed in R using the packages indicated below. Two-sided tests were used with *α* = 0.05. When multiple related endpoints were analyzed (echocardiographic measures), *p*-values were adjusted using the Benjamini–Hochberg (BH) procedure. Echocardiography, cardiomyocyte morphometrics, and proliferation data were analyzed using the *glmmTMB*^47^ package, and model diagnostics were assessed with *DHARMa*^48^. Echocardiographic parameters were modeled as Gaussian mixed-effects models in *glmmTMB*, treating group and age as fixed factors and animal as a random factor; *p*-values for the main effects were obtained by likelihood-ratio tests and adjusted using the BH procedure across endpoints. For nested-design experiments (proliferation and morphometrics), *p*-values were derived from estimated marginal means using *emmeans*^49^ with a conservative degrees-of-freedom estimate (d*f* = *n*₁ + *n*₂ − 2), as asymptotic z-tests from *glmmTMB* can produce overly optimistic results when the number of animals is smaller relative to the number of measurements. Cardiomyocyte morphometric shape parameters were modeled with *glmmTMB*, where the shape variable was expressed as a function of Genotype and Animal was treated as a random factor, with one observation corresponding to one cardiomyocyte. Cardiomyocyte length, width, and area were modeled using a Gamma distribution with a log link, whereas eccentricity was logit-transformed and modeled with a t-distribution (identity link). Proliferation rates of embryonic cardiomyocytes were modeled using a Conway–Maxwell–Poisson regression including the full factorial of genotype and developmental stage, with animal as a random factor and total cell number included as an offset. Survival data (days after birth, control vs. cKO) were analyzed by fitting Kaplan–Meier curves and comparing groups with a log-rank test using the *survival*^50,51^ package. For non-echocardiography endpoints, sample processing and quantification were performed using coded identifiers where feasible; allocation was genotype-defined. All reported *n* values represent biological replicates (animals/embryos), technical replicates, and nested measurements (cells/fields/sections) were accounted for by mixed-effects modeling as described above. No statistical methods were used to predetermine sample size; sample sizes were chosen based on prior experience with these assays and feasibility constraints. No animals/embryos were excluded from analyses. Whole-mount, histology, and immunofluorescence images shown are representative of consistent observations across multiple independent embryos/animals and litters.

### Reporting summary

Further information on research design is available in the Nature Portfolio Reporting Summary linked to this article.

## Supplementary information

Supplementary Information

Description of Additional Supplementary Materials

Supplementary Data 1

Reporting Summary

Transparent Peer Review File

## Data Availability

The bulk RNA-seq data generated in this study have been deposited in the NCBI Gene Expression Omnibus under accession GSE311541. The mass spectrometry proteomics data, including TMT-based proteome and phosphoproteome datasets, have been deposited in MASSIVE (MSV000100933) and ProteomeXchange (PXD074727). Processed datasets and numerical source data underlying the figures, together with custom analysis scripts, have been deposited on Figshare^52^. All other data supporting the findings of this study are available from the corresponding author on reasonable request.

## Code availability

Custom R/Quarto analysis scripts for ventricular shape analysis, longitudinal echocardiography, and embryonic cardiomyocyte proliferation have been deposited on Figshare^52^. Software versions and parameters are detailed in the “Methods” section.
