# Supplementary material for: A narrow developmental window defines PKN2’s essential role in ventricular chamber morphogenesis
Source: Commun Biol. 2026 May 16;9:1042. doi: 10.1038/s42003-026-10287-9 (PMC13439452; doi:10.1038/s42003-026-10287-9)
Supplement: Supplementary file 1 [file 42003_2026_10287_MOESM1_ESM.pdf]

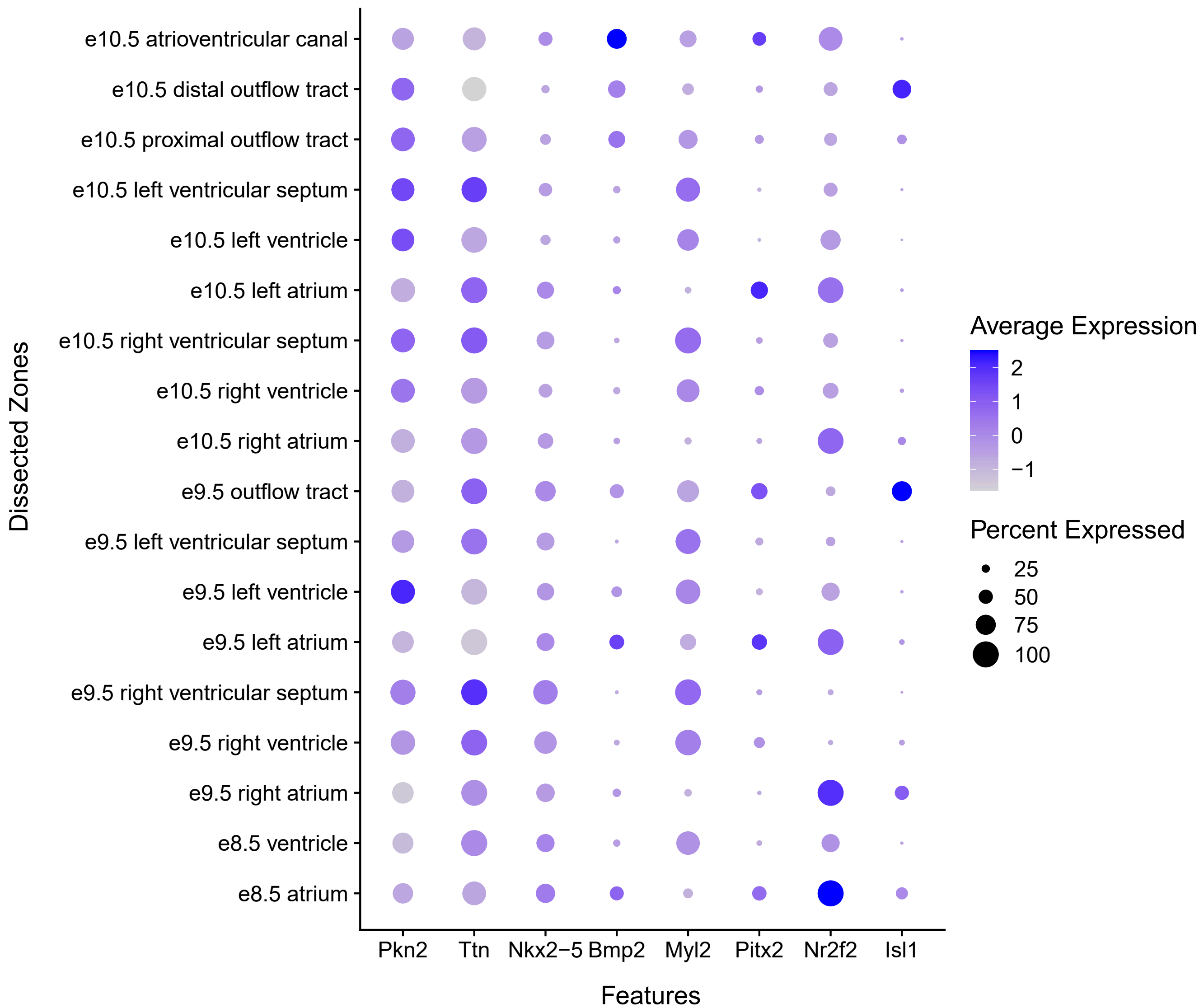

Figure S1. Single-cell expression of Pkn2 during embryonic heart development (GSE76118; PMID: 27840109). Cells are grouped by dissected anatomical region and developmental stage. Color denotes mean expression; dot size denotes fraction of expressing cells. Reference region-specific genes are included as internal validation controls.

Methods. Raw per-cell gene read count files for the embryonic mouse heart dataset GSE76118 were downloaded from GEO and merged into a gene-by-cell count matrix in R. Gene identifiers were annotated by matching Ensembl gene IDs to gene names using a curated annotation table. The count matrix was analyzed in Seurat, and cells were retained after quality control using thresholds of 1,500–12,500 detected genes, 4×10^5–5×10^6 total counts, and ≤20% mitochondrial reads. Data were processed using a standard Seurat workflow, including normalization, scaling, principal component analysis, clustering, and UMAP visualization. For the dot plot, single cells were grouped by dissected anatomical zones and developmental time points, and gene expression was summarized within each group.

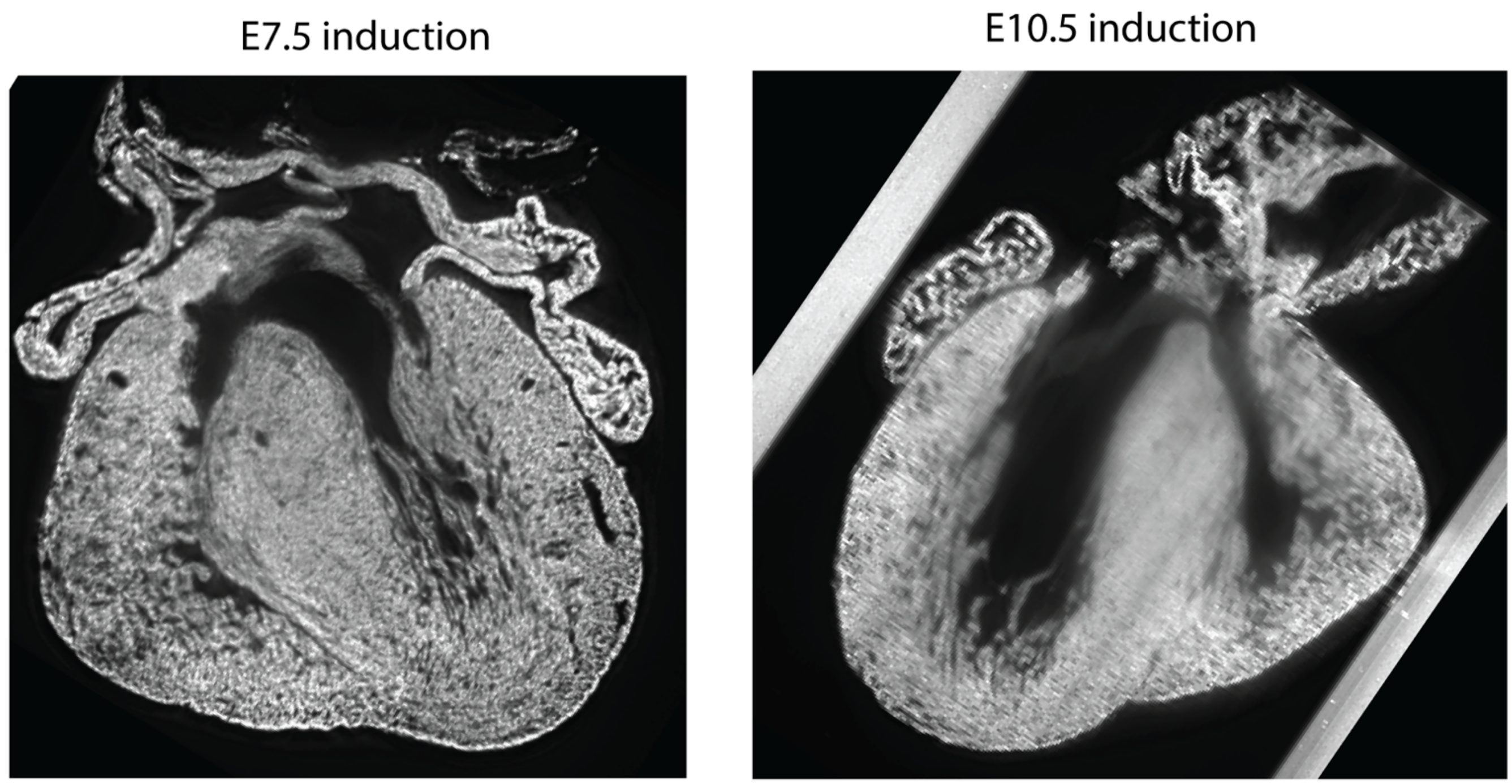

Figure S2. Uniform mT/mG reporter labeling after Tnnt2MerCreMer induction at E7.5 or E10.5. Representative single-plane optical sections from cleared, light-sheet imaged hearts carrying the $Rosa26^{mT/mG}$ reporter (mG channel). Membrane-targeted GFP labeling is widespread throughout the ventricular myocardium following tamoxifen induction at either E7.5 or E10.5, indicating efficient recombination across the ventricular wall.

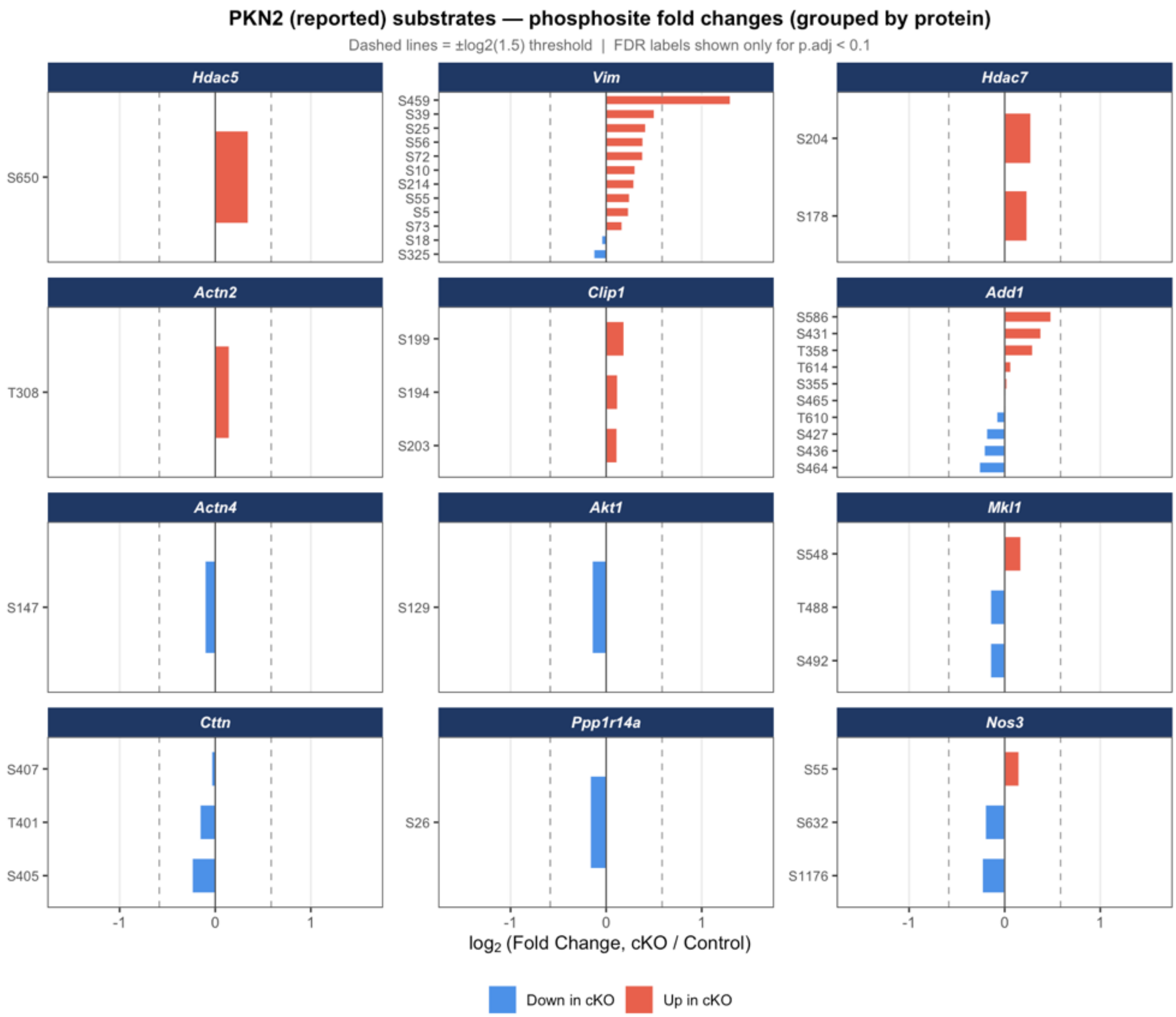

Figure S3. Phosphosite changes on reported PKN2 substrate proteins in E10.5 hearts. Phosphoproteomic fold-changes ($\log_2$ cKO/Control) for phosphopeptides mapping to literature-reported PKN2 substrate proteins. Each panel represents one substrate protein, with individual bars corresponding to detected phosphosites. Red bars indicate increased phosphorylation in PKN2 cKO hearts; blue bars indicate decreased phosphorylation.

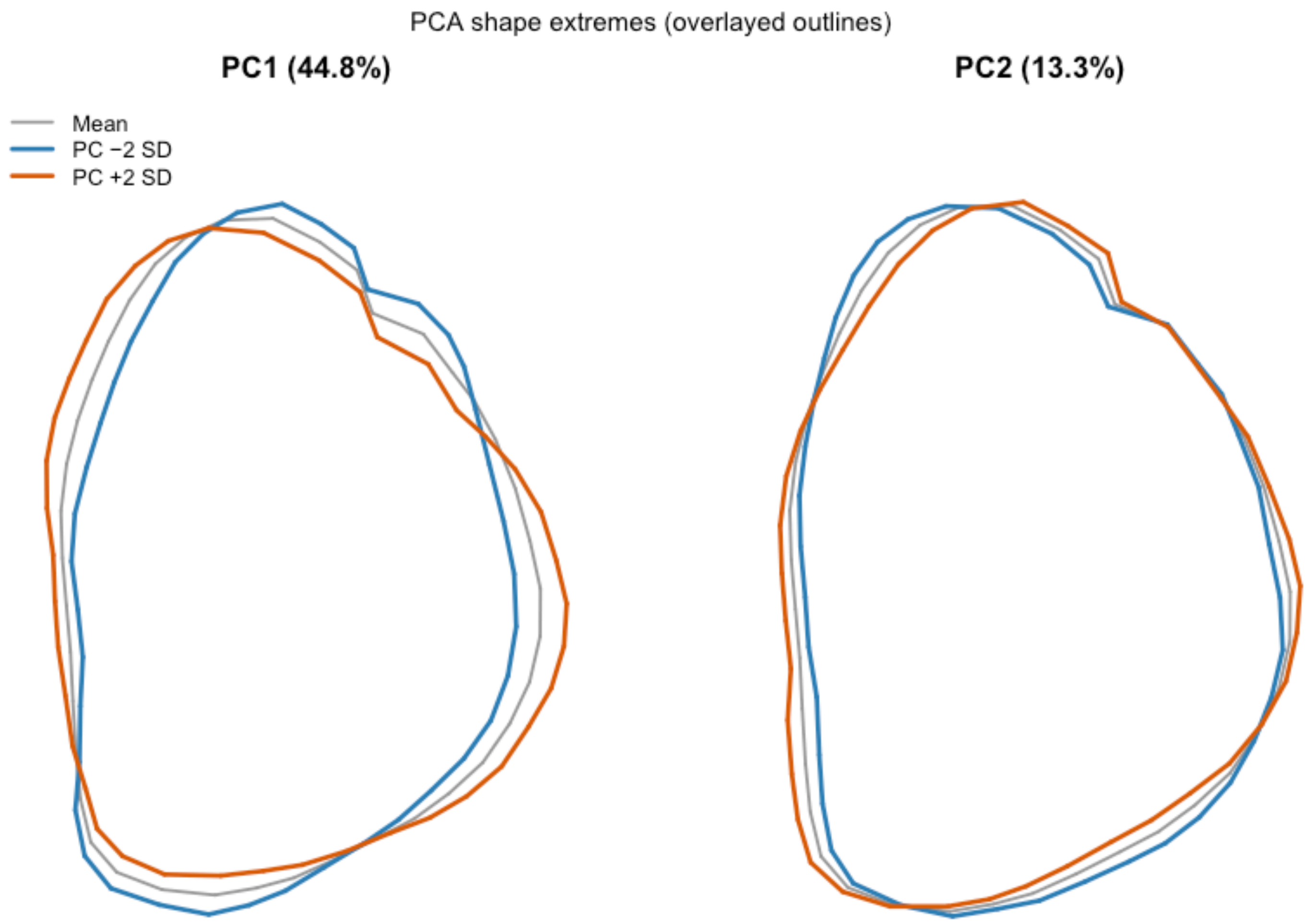

Figure S4. Shape extremes of principal components describing ventricular geometry. Overlay of the mean ventricular contour (grey) with ±2 SD shape extremes for PC1 and PC2 derived from Procrustes-aligned semilandmarks. PC1 (44.8% of variance) captures the dominant change in ventricular geometry, spanning elongated contours (−2 SD) to broader, laterally expanded "coin-pouch" shapes (+2 SD). PC2 (13.3%) represents a secondary mode of contour displacement. Notably, ventricles from E7.5-induced Pkn2 cKO hearts project toward the positive side of PC1, corresponding to the broadened contour shown at +2 SD, illustrating that the principal morphometric axis captures the characteristic geometry of the early-induction phenotype.

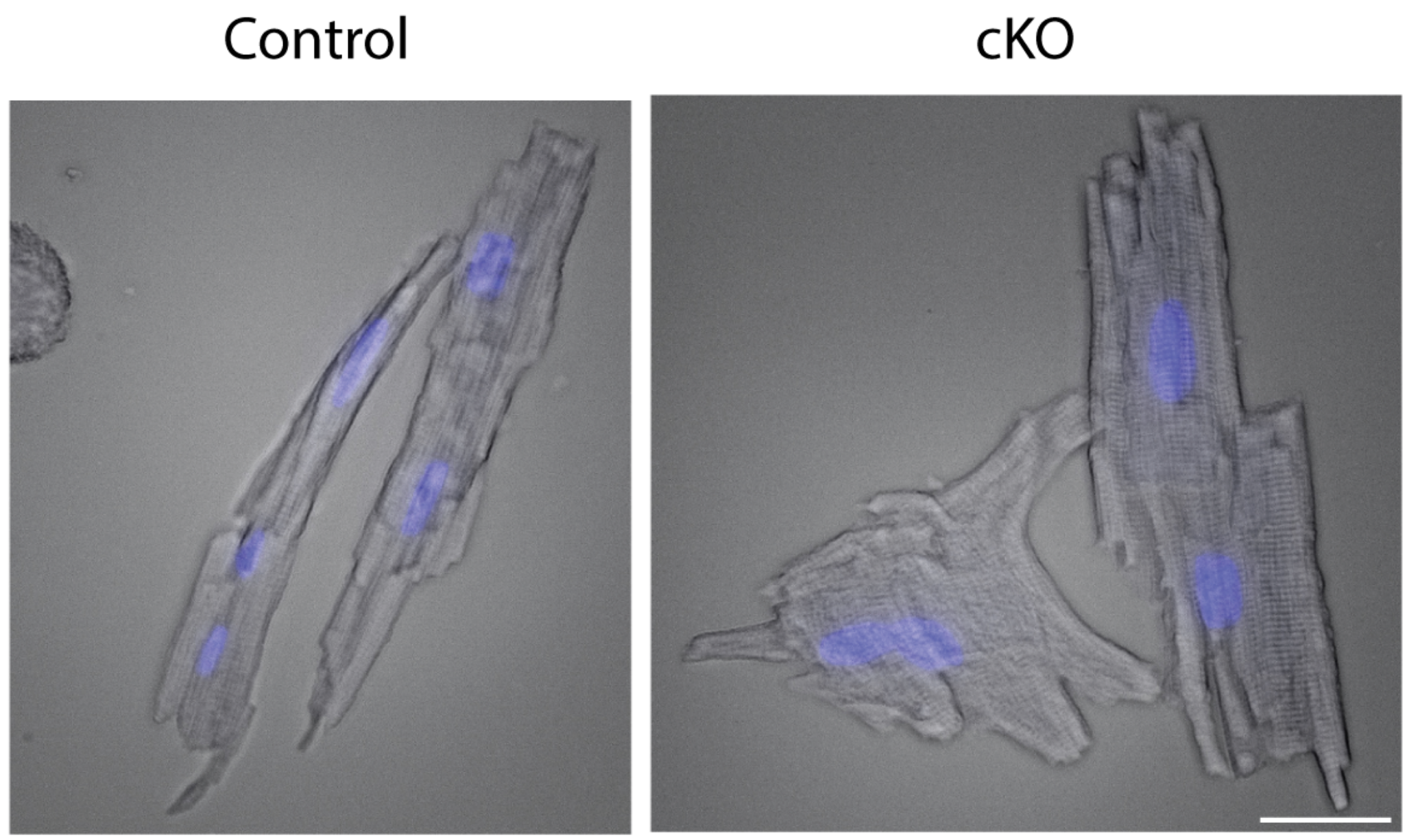

Figure S5. Representative isolated cardiomyocytes used for morphometric analysis. Representative ventricular cardiomyocytes from control and Pkn2 cKO hearts used for the quantitative analysis shown in Figure 1D. Nuclei are indicated (blue). Consistent with the quantitative results, cKO cardiomyocytes appear thicker. Scale bar, 20 μm.
