# Supplementary material for: A narrow developmental window defines PKN2’s essential role in ventricular chamber morphogenesis
Source: Commun Biol. 2026 May 16;9:1042. doi: 10.1038/s42003-026-10287-9 (PMC13439452; doi:10.1038/s42003-026-10287-9)
Supplement: Supplementary file 2 [file 42003_2026_10287_MOESM2_ESM.pdf]

Description of Additional Supplementary Files

File name: Supplementary Data 1

Description: PKN2 (PRK2) — Direct Protein Kinase Substrates
