# Supplementary material for: A narrow developmental window defines PKN2’s essential role in ventricular chamber morphogenesis
Source: Commun Biol. 2026 May 16;9:1042. doi: 10.1038/s42003-026-10287-9 (PMC13439452; doi:10.1038/s42003-026-10287-9)
Supplement: Supplementary file 4 [file 42003_2026_10287_MOESM5_ESM.pdf]

nature portfolio

Peer Review File

A narrow developmental window defines PKN2's essential role in ventricular chamber morphogenesis

Corresponding Author: Professor Ju Chen

This file contains all reviewer reports in order by version, followed by all author rebuttals in order by version.

Version 0:

Reviewer comments:

Reviewer #1

(Remarks to the Author)
The paper shows that cardiomyocyte PKN2 is required only during an early, already well-defined proliferative/migratory window for proper ventricular morphogenesis, and that later deletion is largely tolerated; mechanistically it reiterates PKN2's generic role in coordinating cytoskeletal dynamics and proliferation.
Summary
The authors use XMLC2-Cre;Pkn2fl/fl mice to delete Pkn2 in cardiomyocytes, describing partial early postnatal lethality, non-progressive systolic dysfunction, and a "coin-pouch" ventricular geometry that resembles an embryonic heart shape. Developmental staging shows that hearts appear grossly normal at E10.5 but diverge in surface geometry and external contour by E11.5–E14.5, prompting multi-omic profiling at E10.5 (RNA-seq, proteomics, phosphoproteomics). At E10.5, gene-by-gene changes are modest, but GSEA highlights upregulation of actin cytoskeleton/motility programs and downregulation of chromosome-condensation/mitotic modules, which the authors interpret as a coordinated shift in cytoskeletal and proliferative state. An inducible Tnnt2-MerCreMer;Pkn2fl/fl model with tamoxifen at E7.5 versus E10.5, coupled to light-sheet–based 3D morphometrics, shows that early (E7.5) but not later (E10.5) cardiomyocyte deletion reproduces the abnormal ventricular geometry. EdU/NKX2.5 labelling demonstrates reduced cardiomyocyte proliferation at E10.5–E11.5 in PKN2 cKO hearts, consistent with the "condensed chromosome" GSEA signal, and the Discussion situates these findings within the known E9.5–E12.5 clonal growth/motility window and prior PKN2 work in fibroblasts and mesoderm.

Major criticisms:
Conceptual and novelty
Incremental extension of prior PKN2 cardiac knockout studies: Earlier XMLC2-Cre;Pkn2fl/fl work already established that cardiomyocyte PKN2 is indispensable for ventricular chamber formation, with diverticula, VSDs, thin compact myocardium, and high lethality. This manuscript largely refines the timing and penetrance of that same phenotype on a slightly different background, rather than uncovering a qualitatively new role or pathway.
Developmental window is expected, not surprising: The conclusion that PKN2 is essential only during an early phase of high proliferation and motility (before roughly E10.5–E12.5) aligns directly with established clonal analyses that define this same window as the period of cytoskeleton-dependent ventricular wall growth. Showing that a cytoskeleton/proliferation regulator follows the same window, and then becomes dispensable, is conceptually predictable rather than genuinely novel.
Mechanistic insight remains generic: Multi-omics and EdU data confirm that PKN2 controls cardiomyocyte proliferation and actin-based behavior, but this mirrors its known roles in fibroblasts and mesoderm, where PKN2 regulates cytoskeletal organization, cell migration, and cell-cycle progression. The study does not identify specific downstream effectors, unique cardiac interactors, or pathway-level mechanisms that would advance understanding beyond "PKN2 coordinates cytoskeleton and proliferation in cardiomyocytes, as in other cell types."
Experimental/interpretation
GSEA with subtle primary changes: At E10.5, the authors report "few large-effect hits" in any modality and rely heavily on GSEA of modest rank-based shifts to claim coordinated induction of actin programs and repression of mitotic modules. Without strong per-gene or per-protein effects or validation of key candidates, these enrichment patterns risk being descriptive rather than mechanistically informative.
Morphometrics emphasize shape quantification, not biological mechanism: The light-sheet/Procrustes pipeline convincingly quantifies shape differences between early and late induction, but it does not dissect which specific cellular processes (directional migration vs. oriented division vs regional proliferation) are altered. As a result, a technically sophisticated

morphometric analysis is used primarily to restate that “early knockout has a bigger effect on shape than late knockout,” which is already apparent qualitatively.
Limited integration with human relevance: Although the Introduction notes human genetic associations of PKN2 with myocardial infarction and arrhythmias, the study does not connect the embryonic timing window or the “coin-pouch” geometry to any specific human congenital phenotype, variant, or mechanism. The work thus remains an internally consistent mouse developmental story with relatively weak translational or disease-mechanistic impact.
Minor criticisms
The authors use a previously available Tnnt2MerCreMer mouse line to regulate the temporal window of PKN2 deletion. There is no reference for this line in the paper, presumably is the one published in doi:10.1002/dvg.22861. Although tamoxifen is administered at E7.5 and E10.5 to assess temporal requirements, Tnnt2 is only weakly expressed at E7.5. Given the 10–12 h delay required for tamoxifen-dependent Cre activity, effective PKN2 inactivation is likely to occur no earlier than E8.5.

Reviewer #2

(Remarks to the Author)
Bogomolovas et al have studied the role of the protein kinase PKN2 in ventricular development. While a number of earlier studies have identified the importance of PKN2 in the heart this careful study refines understanding of the role of this protein in ventricular development. The authors use conditional loss of function experiments to define an early developmental time window of myocardial PKN2 activity that is essential for later ventricular form and function. The following points need to be addressed to reinforce the conclusions.

1. Can the authors demonstrate that the early induction of the Cre line is restricted to cardiomyocytes and doesn't remove PKN2 activity in progenitor cells? Please provide data as to the recombination efficiency and labelling using the included mTmG reporter, comparing early and late Cre induction.

2. Please provide information concerning the expression of Pkn2 during the early time window, based on transcript or protein expression data.

3. Concerning the quantitative analysis, the authors should elaborate in the results on how the semilandmarks were defined and the nature of the two principal components that capture the shape abnormalities of the mutant hearts. Can the authors provide any quantitative morphometric analysis of constitutive KO hearts to compare with conditional mutants hearts induced during the early time window?

4. It is unclear whether the phosphoproteomic analysis revealed any potentially direct PKN2 target proteins. The authors need to provide more data from these experiments that may yield insights into the mechanisms underlying the phenotype.

5. In Figure 2 the grey, red and blue key under panel B is confusing - does the red really indicate PKN2? Or significantly downregulated genes/proteins/phosphoproteins? Linked with previous point, this data should be accompanied by a series of tables listing the identified molecules.

6. Please clarify what is meant by active cardiomyocyte migration. Do the authors consider this to be a morphogenetic defect rather than one of differentiation?

7. Please indicate the ventricular diverticulum and the cardiomyocyte disarray in Figure 1 panel C as well as phenotypic features referred to in the results in Figure 2 panel A.

8. It would be helpful to show examples of labelled cardiomyocytes to accompany Figure 1 panel D.

Minor point

9. Please provide the address for affiliation 2.

Reviewer #3

(Remarks to the Author)
Manuscript: COMMSBIO-25-10823

"A narrow developmental window defines PKN2's essential role in ventricular chamber morphogenesis", Bogomolovas et al.

Brief summary of the manuscript
This manuscript examines the role of Protein Kinase N2 (PKN2) in embryonic heart development using cardiomyocyte-specific constitutive and inducible knockout mouse models. By combining developmental staging, multi-omics profiling at a pre-phenotypic stage (E10.5), cardiomyocyte proliferation assays, and quantitative 3D ventricular morphometrics, Bogomolovas et al. identify a previously unrecognized, temporally restricted requirement for PKN2 during early ventricular morphogenesis. Loss of PKN2 in cardiomyocytes prior to ~E10.5 results in persistent ventricular shape abnormalities and reduced cardiomyocyte proliferation, whereas deletion after this stage largely spares gross ventricular geometry. The study

refines prior genetic work on PKN2 by defining a narrow developmental window during which PKN2 is essential for normal chamber formation.

Overall impression of the work
This work is well focused, technically strong, and conceptually novel. It is a well-executed study that provides a clear conceptual contribution to the field of cardiac developmental biology. The identification of an early, time-limited requirement for PKN2 in cardiomyocytes represents genuine novelty and significantly advances understanding beyond previous studies that established PKN2 as essential but did not resolve when or during which developmental phase this requirement operates.

The experimental strategy is elegant and provides novel insights into early cardiac development and the role of cardiomyocyte PKN2. In particular, the use of inducible cardiomyocyte-specific deletion to causally define developmental timing, coupled with rigorous quantitative ventricular shape analysis, is a major strength. The multi-omics data are well positioned to provide molecular context at a stage preceding overt morphological divergence, strengthening the developmental interpretation without overextending the scope of the study.

Overall, the conclusions are well supported by the data. The manuscript is clearly written, logically organized, and focused. Only minor language refinements are suggested to improve precision and to ensure that claims are carefully aligned with the experimental evidence.

Specific comments, with recommendations

These are minor, language-focused revisions that do not require additional experimentation.

Clarify language related to cardiomyocyte migration
Several passages refer to an “early proliferative and migratory phase” or to disruption of “cell migration.” While the enrichment of cytoskeletal and motility-associated gene sets supports this interpretation, migration is not directly measured in the study.
Recommendation: Slightly soften these statements by emphasizing cytoskeletal remodeling or motility-associated programs rather than explicit migration. This will improve precision without weakening the central conclusions.

Narrow claims regarding dispensability for morphogenesis
Statements indicating that PKN2 becomes dispensable for “heart morphogenesis” or the “remaining morphogenetic program” may read as broader than what was directly assessed.
Recommendation: Specify “gross ventricular morphogenesis” or “ventricular chamber geometry” to align conclusions with the measured outcomes.

Moderate mechanistic phrasing in the Discussion
The multi-omics analysis provides strong molecular context but does not directly establish downstream mechanisms or effectors.
Recommendation: Replace phrases such as “provides a mechanistic rationale” with “provides molecular context” or “identifies molecular programs associated with the phenotype.”

Minor clarity and wording suggestions
Replace “unbiased stage for molecular profiling” with “a stage prior to overt morphological divergence.”
Correct minor typographical issues (e.g., missing words related to postnatal timing).
Ensure consistent use of plural forms (e.g., “gene sets”) and hyphenation (e.g., “multi-omics,” “light-sheet microscopy”).

Version 1:

Reviewer comments:

Reviewer #1

(Remarks to the Author)
This study shows that PKN2 is required in a narrow early developmental window (before ~E10.5) for ventricular morphogenesis. Using constitutive and inducible cardiomyocyte-specific KOs, the authors link early PKN2 loss to abnormal ventricular geometry, reduced cardiomyocyte proliferation, and cytoskeletal gene program changes .
Strengths:
Elegant use of inducible genetics to define timing
Integration of multi-omics with 3D morphometrics
Clear identification of a temporal requirement for PKN2
Major concerns:
1. Limited mechanistic insight. Cytoskeletal involvement is inferred from enrichment analysis; no direct functional evidence (e.g., actin organization, migration, polarity).

2. Overinterpretation of multi-omics data. Few strong individual changes at E10.5, yet strong conclusions are drawn from pathway analysis.
3. Modest proliferation defect. The ~20% reduction may not fully explain the strong morphological phenotype.
4. Temporal window not fully resolved. Only two induction time points (E7.5 vs E10.5) are tested.
5. Potential confounders. Genetic background effects and possible non-cell-autonomous contributions are not fully addressed.
Minor comments:
Clarify statistical methods and sample sizes
Improve figure clarity (especially gene set enrichment plots)
Provide more methodological detail for reproducibility
Impact:
The work refines the temporal role of PKN2 in cardiogenesis and will interest the field, though the advance is incremental without deeper mechanistic insight.

Reviewer #2

(Remarks to the Author)
The authors have addressed my previous points and revised and clarified their manuscript that extends understanding of the role of PKN2 in cardiac development.

Reviewer #4

(Remarks to the Author)
I co-reviewed this manuscript with one of the reviewers who provided the listed reports. This is part of the Communications Biology initiative to facilitate training in peer review and to provide appropriate recognition for Early Career Researchers who co-review manuscripts.

Open Access This Peer Review File is licensed under a Creative Commons Attribution 4.0 International License, which permits use, sharing, adaptation, distribution and reproduction in any medium or format, as long as you give appropriate credit to the original author(s) and the source, provide a link to the Creative Commons license, and indicate if changes were made.
In cases where reviewers are anonymous, credit should be given to 'Anonymous Referee' and the source.
The images or other third party material in this Peer Review File are included in the article's Creative Commons license, unless indicated otherwise in a credit line to the material. If material is not included in the article's Creative Commons license and your intended use is not permitted by statutory regulation or exceeds the permitted use, you will need to obtain permission directly from the copyright holder.
To view a copy of this license, visit https://creativecommons.org/licenses/by/4.0/

Response to Editor:

1. Please provide data on the recombination efficiency / labeling for the reporter lines used (XMLC2-Cre;Pkn2fl/fl and Tnnt2MerCreMer;Pkn2fl/fl), including PKN2 expression data at timepoint analysed.

Thank you for raising this important point. For constitutive cardiomyocyte knockout (XMLC2-Cre;Pkn2$^{fl/fl}$), we used XMLC2-Cre, a pan-myocardial Cre line with early activity in cardiomyocytes. In our hands, efficient recombination/deletion is supported by the robust depletion of Pkn2/PKN2 at the E10.5 timepoint used for molecular profiling. Specifically, Pkn2 is among the most strongly depleted features in our E10.5 datasets across RNA-seq, proteomics, and phosphoproteomics (revised Figure 2B and Supplementary Data), confirming that PKN2 is normally expressed at this stage and is efficiently deleted in the cKO. In addition, to document Pkn2 expression during the relevant developmental window independent of our knockout datasets, we reanalyzed the published embryonic heart single-cell dataset GSE76118 and confirmed that Pkn2 is detected across multiple anatomical regions and developmental stages during early heart morphogenesis. This analysis is now provided as Supplementary Figure S1. For the inducible model (Tnnt2MerCreMer;Pkn2$^{fl/fl}$), all embryos carried the Rosa26$^{mT/mG}$ reporter, and ventricular reconstructions for morphometric analysis were segmented from the membrane-targeted GFP (mG) signal. While robust surface mG labeling is sufficient for accurate surface reconstruction, we agree that a potential concern would be recombination occurring predominantly in superficial layers with incomplete labeling deeper in the ventricular wall. To address this directly, we examined raw optical sections through the ventricle from representative cleared/light-sheet imaged hearts induced at E7.5 and E10.5. At both timepoints, mG labeling is widespread and uniform throughout the ventricular myocardium, with no obvious evidence of a surface-only labeling pattern. We have now included these representative sections as Supplementary Figure S4.

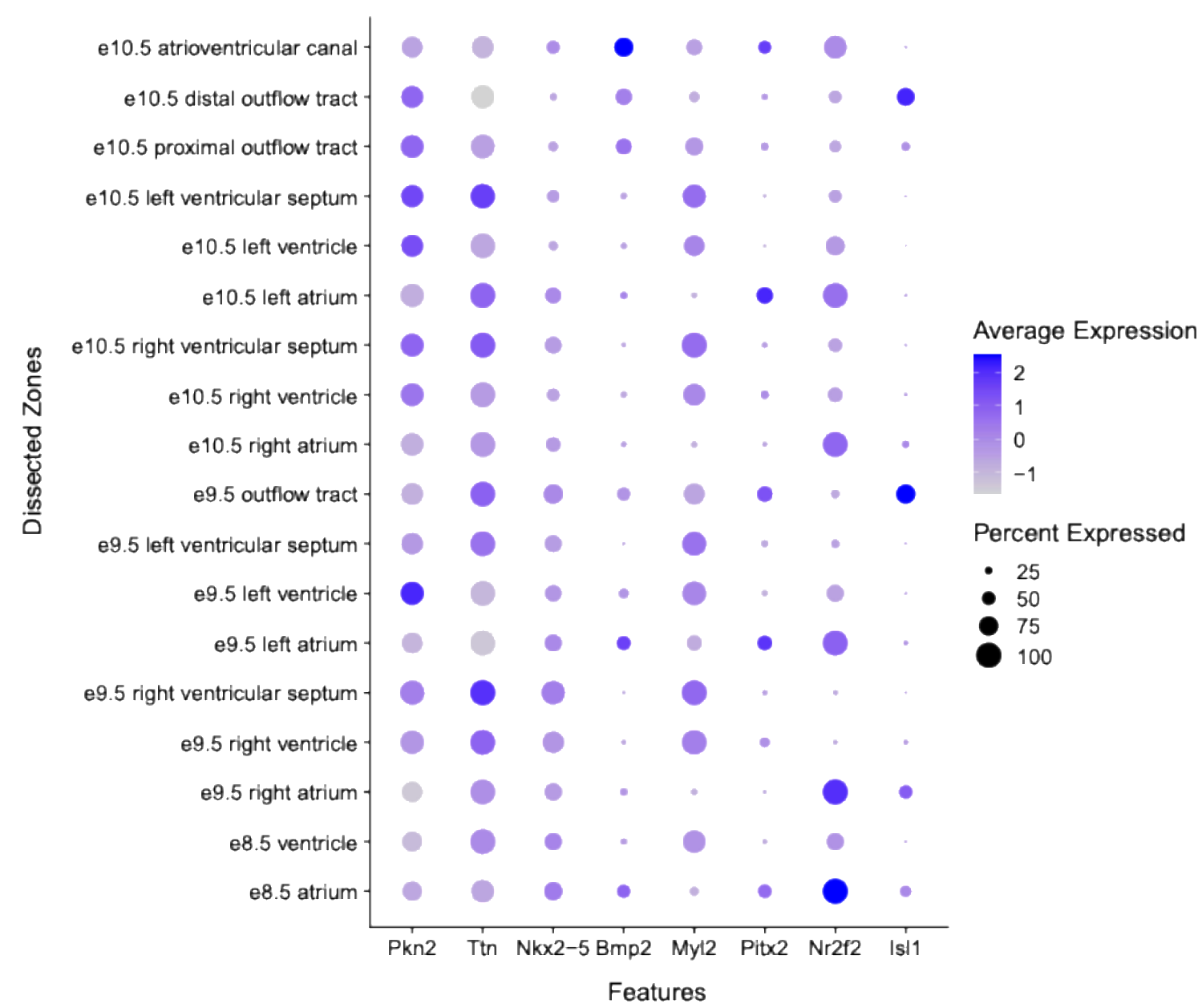

Figure S1. Single-cell expression of Pkn2 during embryonic heart development (GSE76118; PMID: 27840109). Cells are grouped by dissected anatomical region and developmental stage. Color denotes mean expression; dot size denotes fraction of expressing cells. Reference region-specific genes are included as internal validation controls.

2. Investigate if the phosphoproteomic analysis reveals any potentially direct PKN2 target proteins.

We thank you for this important suggestion. To address this question, we curated a list of reported PKN2 substrate proteins from the literature and systematically mapped these onto our E10.5 phosphoproteomics dataset. Phosphopeptides corresponding to multiple reported substrates were detected (including Vim, Add1, Actn family members, Cttn, Clip1, Nos3, Ppp1r14a, Mkl1, Hdac5/7, and Akt1). None of the detected substrate-associated phosphosites reached FDR significance in the global dataset. However, several sites exhibited directional changes in PKN2 cKO hearts, with some sites decreased and others

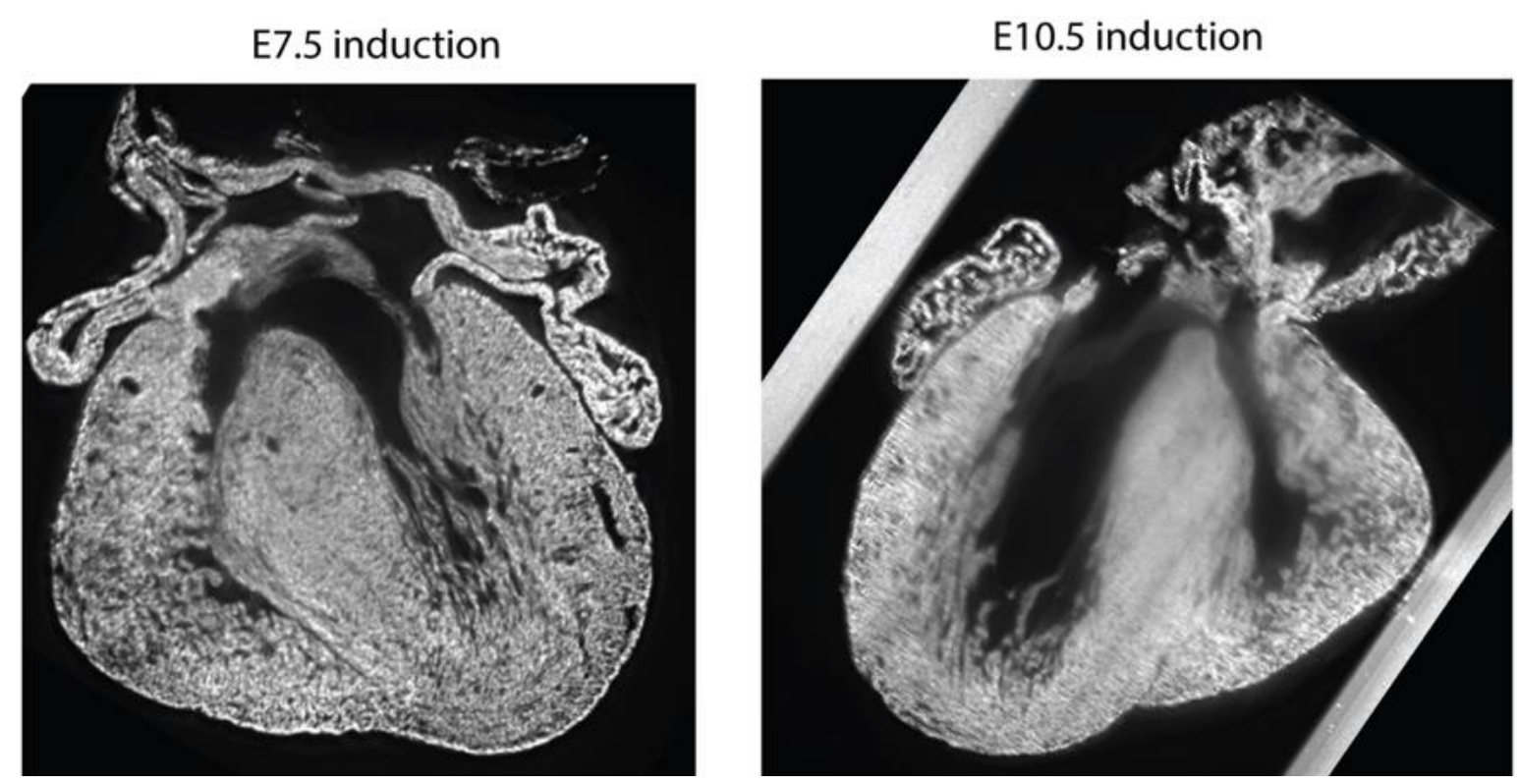

Figure S4. Uniform mT/mG reporter labeling after Tnnt2MerCreMer induction at E7.5 or E10.5. Representative single-plane optical sections from cleared, light-sheet imaged hearts carrying the Rosa26$^{mT/mG}$ reporter (mG channel). Membrane-targeted GFP labeling is widespread throughout the ventricular myocardium following tamoxifen induction at either E7.5 or E10.5, indicating efficient recombination across the ventricular wall.

increased relative to the control. Given the complexity of kinase network compensation and secondary signaling effects in vivo, both up- and down-regulated sites are consistent with PKN2-dependent phosphoregulatory processes rather than strictly direct linear kinase–substrate relationships. We have added this analysis as <u>Supplementary Figure S3</u> and <u>Supplementary Table 1</u> and have incorporated a description of these findings in the Results section of the revised manuscript.

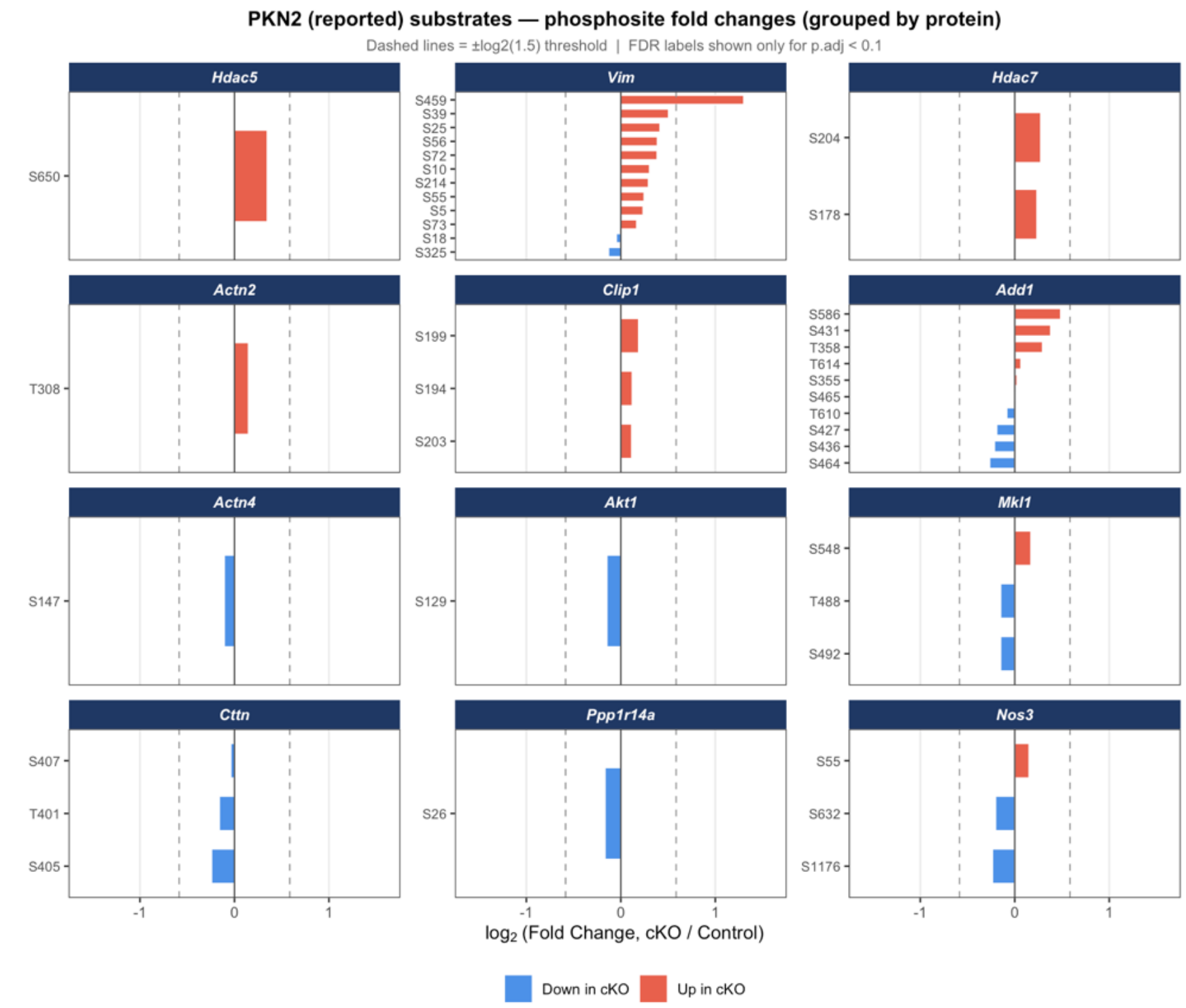

Figure S3. Phosphosite changes on reported PKN2 substrate proteins in E10.5 hearts. Phosphoproteomic fold-changes (log_2 cKO/Control) for phosphopeptides mapping to literature-reported PKN2 substrate proteins. Each panel represents one substrate protein, with individual bars corresponding to detected phosphosites. Red bars indicate increased phosphorylation in PKN2 cKO hearts; blue bars indicate decreased phosphorylation.

3. Provide additional data on specific altered cellular processes, for example directional migration vs. oriented division vs regional proliferation.

We thank the reviewer for this suggestion. To directly address whether specific cellular processes are regionally altered, we quantified cardiomyocyte proliferation separately in the trabecular and compact myocardium at E10.5 and E11.5 using EdU incorporation in NKX2.5^{+} nuclei. As shown in the <u>new Figure 3E</u>, proliferation is significantly reduced in the Pkn2 cKO in both trabecular and compact regions at both developmental stages. These data demonstrate that the defect reflects a broad reduction in regional cardiomyocyte proliferation, rather than a compartment-restricted effect. We have revised the manuscript text accordingly. Because oriented division and directional migration were not directly measured in this study, we have also modified the wording to avoid overinterpretation and now refer specifically to altered proliferation and cytoskeletal/motility-associated programs.

4. For all graphs depicting a single point value (e.g., mean) with error bars, you must add individual data points or convert the graph to a boxplot or dot-plot to show data distribution.

We thank editor for pointing this out. All figures in the revised manuscript are now represented this way.

5. It's mandatory to provide access to the numerical source data for graphs and charts either through a repository or by providing the data in a Supplementary Data file (in excel format).

This has been addressed in the revised manuscript. Numerical data and accompanying R scripts have been uploaded onto Figshare platform: <u>https://figshare.com/account/mycontent/projects/271810</u>

6. All blots/gels must be accompanied by size markers in every figure panel. Uncropped and unedited *blot/gel images must be included as Supplementary Figure(s) in the Supplementary Information pdf.*

Not applicable (no blots/gels are included in this manuscript).

7. Please ensure that you have complied with the data deposition policies at the Nature Portfolio, please see here.

Addressed in the revised manuscript.

8. Please ensure that you have complied with our policies on research involving animals and humans. Please follow the ARRIVE guidelines for reporting animal experiments. Please fully complete an ARRIVE checklist including both the essential and recommended set of items (adding information to the manuscript where needed) and upload this with your revised manuscript.

We have included a completed ARRIVE checklist with the resubmission.

9. Please carefully check your Methods section and ensure that the methods used for all euthanasia, anaesthesia and analgesia procedures of animals are specified (including route and dose as applicable).

We expanded the Methods section accordingly to ensure full compliance with ARRIVE guidelines.

10. Please also see our revision checklist for guidance on formatting the manuscript and complying with our policies. A comprehensive guide to our formatting requirements for final submissions is also available for your reference here.

Addressed in the revised manuscript.

Response to Reviewer #1:

We thank the reviewer for the comment: "The paper shows that cardiomyocyte PKN2 is required only during an early, already well-defined proliferative/migratory window for proper ventricular morphogenesis, and that later deletion is largely tolerated; mechanistically it reiterates PKN2's generic role in coordinating cytoskeletal dynamics and proliferation."

Major criticisms:

1. Conceptual and novelty
Incremental extension of prior PKN2 cardiac knockout studies: Earlier XMLC2-Cre;Pkn2fl/fl work already established that cardiomyocyte PKN2 is indispensable for ventricular chamber formation, with diverticula, VSDs, thin compact myocardium, and high lethality. This manuscript largely refines the timing and penetrance of that same phenotype on a slightly different background, rather than uncovering a qualitatively new role or pathway.

Developmental window is expected, not surprising: The conclusion that PKN2 is essential only during an early phase of high proliferation and motility (before roughly E10.5–E12.5) aligns directly with established clonal analyses that define this same window as the period of cytoskeleton-dependent ventricular wall growth. Showing that a cytoskeleton/proliferation regulator follows the same window, and then becomes dispensable, is conceptually predictable rather than genuinely novel.
Mechanistic insight remains generic: Multi-omics and EdU data confirm that PKN2 controls cardiomyocyte proliferation and actin-based behavior, but this mirrors its known roles in fibroblasts and mesoderm, where PKN2 regulates cytoskeletal organization, cell migration, and cell-cycle progression. The study does not identify specific downstream effectors, unique cardiac interactors, or pathway-level mechanisms that would advance understanding beyond "PKN2 coordinates cytoskeleton and proliferation in cardiomyocytes, as in other cell types."

We appreciate the reviewer's thoughtful assessment. We agree that prior cardiomyocyte-restricted Pkn2 knockout studies (e.g., XMLC2-Cre;Pkn2fl/fl) established that PKN2 is required for ventricular chamber formation. The central advance of our work is to define when cardiomyocyte PKN2 is required during development, which could not be resolved using only constitutive Cre approaches. Using a tamoxifen-inducible, cardiomyocyte-restricted model (Tnnt2MerCreMer;Pkn2fl/fl) combined with quantitative light-sheet 3D morphometrics, we provide causal evidence for a sharp developmental dependency window: early tamoxifen administration (E7.5) recapitulates the ventricular geometry abnormality, whereas later administration (E10.5) largely preserves gross ventricular chamber geometry at E16.5. We have revised the Introduction and Discussion to more explicitly highlight this temporal resolution as the main novelty and to better contextualize differences relative to prior XMLC2-Cre studies (including genetic background effects on penetrance/survival).

While our findings are conceptually consistent with the established E9.5–E12.5 period of high cardiomyocyte proliferation and cytoskeletal remodeling, the temporal boundary and the degree to which PKN2 becomes

dispensable after this transition were not previously tested. Our inducible loss-of-function experiments therefore provide direct, genotype-controlled evidence that cardiomyocytes require PKN2 specifically during this early morphogenetic interval.

Finally, we agree that our multi-omics analysis is not intended to claim a single unique downstream effector. We have therefore moderated mechanistic phrasing throughout and present the omics as molecular context highlighting coordinated pathway-level changes (actin/cytoskeleton- and proliferation-related programs) that precede gross shape divergence. We also increased transparency by providing Supplementary Data and original data for RNA-seq, proteomics, and phosphoproteomics.

2. Experimental/interpretation
GSEA with subtle primary changes: At E10.5, the authors report "few large-effect hits" in any modality and rely heavily on GSEA of modest rank-based shifts to claim coordinated induction of actin programs and repression of mitotic modules. Without strong per-gene or per-protein effects or validation of key candidates, these enrichment patterns risk being descriptive rather than mechanistically informative.

We agree that per-feature changes at E10.5 are modest. We deliberately selected E10.5 because it is a stage prior to overt morphological divergence, where primary molecular shifts are expected to be subtle. For this reason, we used rank-based gene set enrichment analysis and required directional concordance across RNA-seq, proteomics, and phosphoproteomics to identify coherent biological programs, rather than emphasizing individual genes/proteins with large fold changes. To address the reviewer's concern about over-interpretation, we have revised the manuscript to frame these pathway-level enrichments as molecular context associated with the early phenotype, not as definitive mechanistic effectors. Importantly, we connect the down-enrichment of chromosome/mitotic gene sets to an orthogonal in vivo validation: reduced EdU incorporation in NKX2.5+ cardiomyocytes at E10.5–E11.5.

3. Morphometrics emphasize shape quantification, not biological mechanism: The light-sheet/Procrustes pipeline convincingly quantifies shape differences between early and late induction, but it does not dissect which specific cellular processes (directional migration vs. oriented division vs regional proliferation) are altered. As a result, a technically sophisticated morphometric analysis is used primarily to restate that "early knockout has a bigger effect on shape than late knockout," which is already apparent qualitatively.

We agree that the light-sheet morphometrics pipeline provides quantification of ventricular chamber geometry but does not, on its own, discriminate among specific cellular processes such as directional migration, oriented division, or regional proliferation. The primary goal of the morphometrics in our study is to causally define the developmental timing of the requirement for PKN2 using an objective 3D readout, rather than to infer a single cellular mechanism from shape alone. To address this point, we have revised language throughout the manuscript to avoid implying that morphometrics itself resolves the underlying cellular process. In addition, to better connect shape changes to a concrete cellular readout (and to address the editor's related request), we expanded analysis of our existing EdU/NKX2.5 proliferation assay to include regional quantification (e.g., compact vs. trabecular myocardium and/or LV vs. RV) during the critical window (<u>new Figure 3E</u>). Finally, we explicitly state as a limitation that directional migration and division orientation were not directly measured in this study.

4. Limited integration with human relevance: Although the Introduction notes human genetic associations of PKN2 with myocardial infarction and arrhythmias, the study does not connect the embryonic timing window or the "coin-pouch" geometry to any specific human congenital phenotype, variant, or mechanism. The work thus remains an internally consistent mouse developmental story with relatively weak translational or disease-mechanistic impact.

We agree that our study is primarily a mouse developmental biology investigation and does not analyze human patient variants. To strengthen the translational framing without over-claiming, we revised the Introduction and Discussion in three ways:
(i) we clarify the scope of published human genetic associations at the PKN2 locus (largely adult cardiac phenotypes),
(ii) we emphasize that our data provide developmental context for how perturbation of PKN2-dependent cytoskeletal/proliferative programs early in gestation can yield persistent ventricular geometry abnormalities,

and
(iii) we discuss the observed ventricular wall malformations (e.g., diverticula/clefts and compaction defects, with a persistent embryonic-like contour) in the context of congenital ventricular wall disorders, while explicitly noting that our work does not establish a direct causal link to a specific human syndrome.

Minor criticism:

1. The authors use a previously available Tnnt2MerCreMer mouse line to regulate the temporal window of PKN2 deletion. There is no reference for this line in the paper, presumably is the one published in doi:10.1002/dvg.22861. Although tamoxifen is administered at E7.5 and E10.5 to assess temporal requirements, Tnnt2 is only weakly expressed at E7.5. Given the 10–12 h delay required for tamoxifen-dependent Cre activity, effective PKN2 inactivation is likely to occur no earlier than E8.5.

We have added the appropriate citation for the Tnnt2MerCreMer line in the Methods. We also clarified our language around induction timing: throughout the manuscript we now refer to 'tamoxifen administration at E7.5 or E10.5' rather than assuming immediate deletion at those time points, and we explicitly note the expected delay between tamoxifen exposure and Cre-mediated recombination.

Response to Reviewer #2:

We thank the reviewer for the comment: "Bogomolovas et al have studied the role of the protein kinase PKN2 in ventricular development. While a number of earlier studies have identified the importance of PKN2 in the heart this careful study refines understanding of the role of this protein in ventricular development. The authors use conditional loss of function experiments to define an early developmental time window of myocardial PKN2 activity that is essential for later ventricular form and function."

1. Can the authors demonstrate that the early induction of the Cre line is restricted to cardiomyocytes and doesn't remove PKN2 activity in progenitor cells? Please provide data as to the recombination efficiency and labelling using the included mTmG reporter, comparing early and late Cre induction.

We would like to emphasize that in inducible KO experiments all embryos carried the Rosa26mT/mG reporter, and ventricular reconstructions were segmented from the membrane GFP (mG) signal, i.e., the post-recombination label. To make this explicit and address the concern that labeling could be superficial, we now include additional optical sections (<u>Supplementary Figure S2</u>; see response to editor) showing that mG labeling extends throughout the ventricular myocardium after both E7.5 and E10.5 induction, rather than being confined to the surface. While we did not lineage-trace progenitors directly, we used the Figure 3 dataset to test whether early, constitutive cardiomyocyte deletion of PKN2 could be associated with a loss of CM progenitors resulting in changes of cardiomyocyte numbers in later developmental stages. Specifically, we compared total cardiomyocytes (NKX2.5$^+$ nuclei) relative to total cells (DAPI$^+$ nuclei) across stage (E10.5 vs. E11.5) and region (compact vs non-compact). A mixed-effects count model with a log (total cells) offset showed no significant genotype effect on cardiomyocyte numbers (Type II Wald test: χ^2=2.68, df=1, p=0.102), and no genotype interactions with stage or area (all p≥0.169; genotype×area p=0.975). Together, these findings support robust myocardial recombination in the inducible model and argue against a detectable change in cardiomyocyte numbers due to early loss of PKN2.

2. Please provide information concerning the expression of Pkn2 during the early time window, based on transcript or protein expression data.

Thank you for this suggestion. We have added transcript-level evidence that Pkn2 is expressed during the relevant early developmental window. Specifically, we reanalyzed the publicly available embryonic mouse heart single-cell RNA-seq dataset GSE76118 and generated an anatomically indexed dot plot showing Pkn2 expression across dissected heart regions and developmental stages. As positive controls for the reanalysis pipeline, we include several genes reported in the original study to exhibit region- and stage-specific expression. This new analysis demonstrates that Pkn2 transcripts are detectable across myocardial-containing anatomical zones during the early window examined in our study. The revised manuscript now includes the new dot plot (<u>new Supplementary Figure S1</u>) and a corresponding subsection detailing the reanalysis workflow. In addition, our E10.5 proteomic and phosphoproteomic datasets detect PKN2 in control hearts and show the expected depletion in cKO hearts, supporting the presence of PKN2 protein at the profiled stage.

3. Concerning the quantitative analysis, the authors should elaborate in the results on how the semilandmarks were defined and the nature of the two principal components that capture the shape abnormalities of the mutant hearts. Can the authors provide any quantitative morphometric analysis of constitutive KO hearts to compare with conditional mutants hearts induced during the early time window?

We thank the reviewer for this helpful suggestion. We have expanded the Results section to better describe how semilandmarks were defined and how the principal components relate to ventricular shape variation. Briefly, ventricular geometry was quantified using 50 sliding semilandmarks placed along a homologous contour of the dorsal ventricular ridge, which were transferred across specimens using a template-based approach (ALPACA) and subsequently allowed to slide to optimize geometric correspondence prior to generalized Procrustes alignment. To further clarify the biological meaning of the principal components, we now include Supplementary Figure S5, which shows the ±2 SD shape extremes for PC1 and PC2. These visualizations illustrate how PC1 primarily reflects global ventricular geometry (elongated versus broader "coin-pouch" contours), whereas PC2 captures more subtle regional changes in the ventricular outline. The extremes closely mirror the qualitative shape differences observed between control and early PKN2-deficient hearts, helping to link the morphometric axes with the anatomical phenotype. Regarding the reviewer's suggestion to include morphometric analysis of constitutive cardiomyocyte Pkn2 knockout hearts, we agree that such a comparison would be informative. However, the light-sheet morphometric dataset used here was generated specifically from the inducible deletion experiments designed to define the temporal requirement for PKN2. Constitutive knockout hearts were not processed through the same clearing, imaging, and semilandmark pipeline and therefore could not be directly incorporated into the same quantitative analysis. Nevertheless, the ventricular geometry observed following early inducible deletion (E7.5) closely phenocopies the morphology described for constitutive Pkn2 cKO hearts, supporting the interpretation that the early developmental window identified in the inducible model corresponds to the stage at which the constitutive phenotype arises.

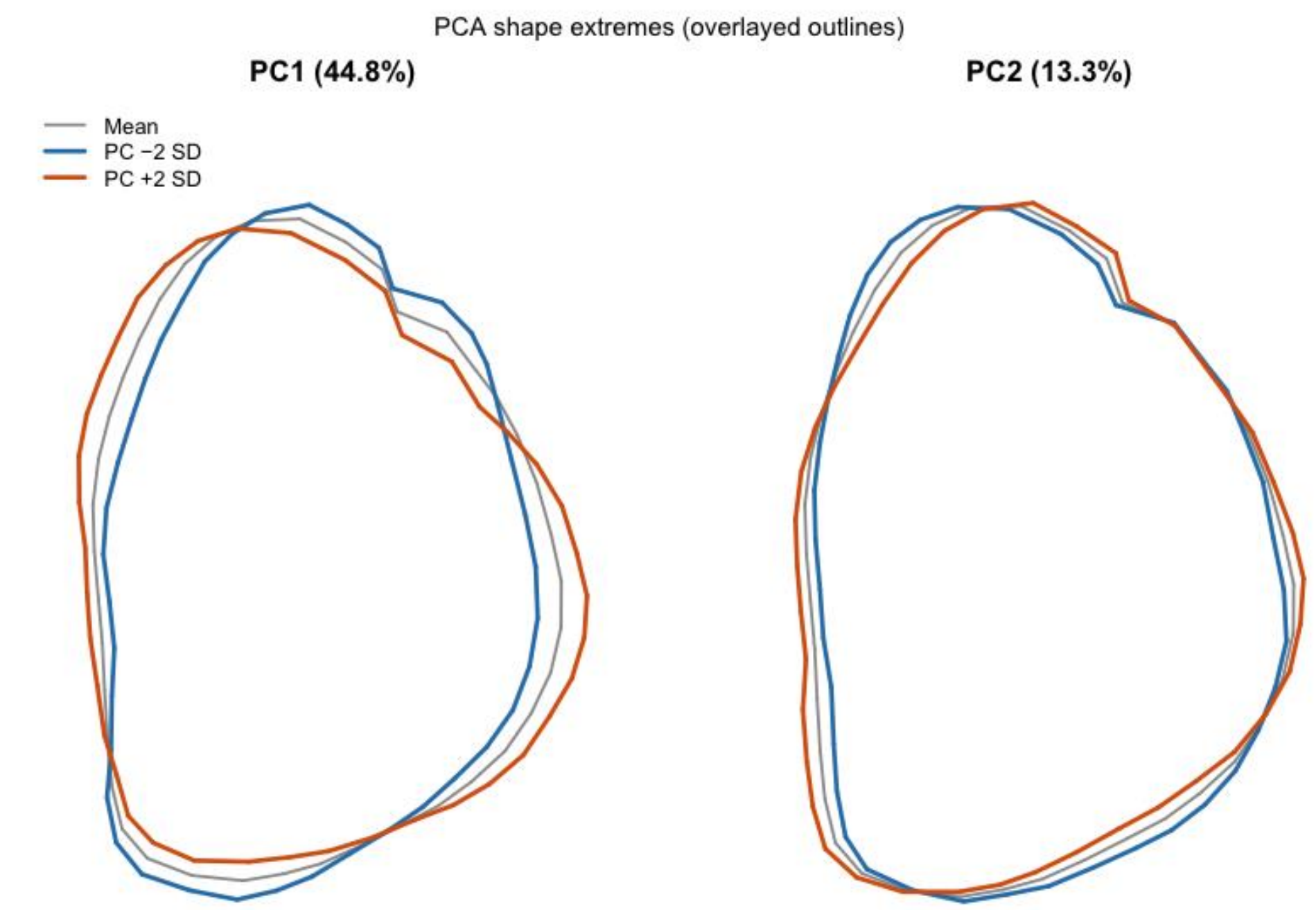

Figure S5. Shape extremes of principal components describing ventricular geometry. Overlay of the mean ventricular contour (grey) with ±2 SD shape extremes for PC1 and PC2 derived from Procrustes-aligned semilandmarks. PC1 (44.8% of variance) captures the dominant change in ventricular geometry, spanning elongated contours (−2 SD) to broader, laterally expanded "coin-pouch" shapes (+2 SD). PC2 (13.3%) represents a secondary mode of contour displacement. Notably, ventricles from E7.5-induced Pkn2 cKO hearts project toward the positive side of PC1, corresponding to the broadened contour shown at +2 SD, illustrating that the principal morphometric axis captures the characteristic geometry of the early-induction phenotype.

4. It is unclear whether the phosphoproteomic analysis revealed any potentially direct PKN2 target proteins. The authors need to provide more data from these experiments that may yield insights into the mechanisms underlying the phenotype.

We thank the reviewer for this important comment. To determine whether our phosphoproteomic dataset contains evidence of potentially direct PKN2 targets, we curated a list of reported PKN2 substrates from the literature and mapped these systematically onto our E10.5 phosphoproteomics data. Phosphopeptides corresponding to multiple reported substrates - including Vimentin, Add1, Actn family members, Cttn, Clip1, Nos3, Ppp1r14a, Mkl1, Hdac5/7, and Akt1 - were detected in our dataset and are now summarized in

Supplementary Figure S3 and Supplementary Table 1. Although none of these substrate-associated phosphosites reached FDR significance in the global comparison, several exhibited consistent directional changes in PKN2 cKO hearts. Notably, altered phosphorylation was observed in several cytoskeleton-associated proteins, which is concordant with the actin/cytoskeletal enrichment identified independently in the transcriptomic and proteomic analyses. Given the complexity of kinase signaling in vivo and the likelihood of compensatory network effects following genetic deletion, we do not expect a simple one-to-one loss of individual phosphosites. Instead, the presence of known PKN2 substrates within the dataset, together with coordinated phosphorylation changes in cytoskeletal regulators and parallel multi-omic enrichment of actin-associated programs, supports a model in which disruption of PKN2-dependent phosphoregulation contributes to the early morphogenetic phenotype. These analyses have been added to the revised manuscript and are presented in Supplementary Figure S3 and Supplementary Table 1.

5. In Figure 2 the grey, red and blue key under panel B is confusing - does the red really indicate PKN2? Or significantly downregulated genes/proteins/phosphoproteins? Linked with previous point, this data should be accompanied by a series of tables listing the identified molecules.

We thank the reviewer for pointing out the ambiguity in the color key. We have revised the Figure 2 legend to clearly define the color coding. In the updated figure, the red point highlights the targeted gene/protein (Pkn2/PKN2), blue points indicate statistically significant differentially regulated features, and gray points indicate non-significant features. In the phosphoproteomics dataset, two red-highlighted points correspond to independent phosphopeptides derived from PKN2, which further supports robust detection of PKN2 depletion at the phosphosite level. We intentionally highlight Pkn2/PKN2 to visually confirm that the targeted kinase is consistently among the most strongly depleted entities across all three modalities (RNA-seq, proteomics, and phosphoproteomics), providing an internal validation that the genetic perturbation is effectively captured at the molecular level. As requested, we now provide tables listing all detected molecules and differential analysis results for each omics layer (RNA-seq, proteomics, and phosphoproteomics) as Supplementary Data files. In addition, to improve transparency and reproducibility, we include the processed tables used to generate the volcano plots and enrichment analyses.

6. Please clarify what is meant by active cardiomyocyte migration. Do the authors consider this to be a morphogenetic defect rather than one of differentiation?

We agree that the phrasing around “active cardiomyocyte migration” was too strong. Our intent was to refer to the early developmental phase characterized by cytoskeletal remodeling and motility-associated programs, as supported by pathway enrichment, rather than to claim that we directly measured migration in vivo. We have therefore revised the manuscript to replace “migration/migratory” wording with more precise language (e.g., “motility-associated cytoskeletal programs” and “cytoskeletal remodeling”). We also clarify in the Results and Discussion that our direct cellular readout is reduced cardiomyocyte proliferation (EdU/NKX2.5) during the critical window, and that we interpret the phenotype primarily as a defect in ventricular morphogenesis/chamber shaping rather than cardiomyocyte differentiation.

7. Please indicate the ventricular diverticulum and the cardiomyocyte disarray in Figure 1 panel C as well as phenotypic features referred to in the results in Figure 2 panel A.

We have updated the figures as requested. In the revised Figure 1C we now annotate the ventricular diverticulum/cleft and regions of cardiomyocyte disarray, and in Figure 2A we added labels indicating the key phenotypic features described in the Results.

8. It would be helpful to show examples of labelled cardiomyocytes to accompany Figure 1 panel D.

We agree that representative examples would help illustrate the cardiomyocyte morphology underlying the quantitative analysis in Figure 1D. We have therefore added representative images of isolated cardiomyocytes from control and Pkn2 cKO hearts, including example nuclear labeling as Supplementary Figure S2.

Minor point:

1. Please provide the address for affiliation.

We have added the full institutional address for affiliation 2 in the author affiliation list in the revised manuscript.

Response to Reviewer #3:

We thank the reviewer for the comments: "This manuscript examines the role of Protein Kinase N2 (PKN2) in embryonic heart development using cardiomyocyte-specific constitutive and inducible knockout mouse models. By combining developmental staging, multi-omics profiling at a pre-phenotypic stage (E10.5), cardiomyocyte proliferation assays, and quantitative 3D ventricular morphometrics, Bogomolovas et al. identify a previously unrecognized, temporally restricted requirement for PKN2 during early ventricular morphogenesis. Loss of PKN2 in cardiomyocytes prior to ~E10.5 results in persistent ventricular shape abnormalities and reduced cardiomyocyte proliferation, whereas deletion after this stage largely spares gross ventricular geometry. The study refines prior genetic work on PKN2 by defining a narrow developmental window during which PKN2 is essential for normal chamber formation." and "This work is well focused, technically strong, and conceptually novel. It is a well-executed study that provides a clear conceptual contribution to the field of cardiac developmental biology. The identification of an early, time-limited requirement for PKN2 in cardiomyocytes represents genuine novelty and significantly advances understanding beyond previous studies that established PKN2 as essential but did not resolve when or during which developmental phase this requirement operates. The experimental strategy is elegant and provides novel insights into early cardiac development and the role of cardiomyocyte PKN2. In particular, the use of inducible cardiomyocyte-specific deletion to causally define developmental timing, coupled with rigorous quantitative ventricular shape analysis, is a major strength. The multi-omics data are well positioned to provide molecular context at a stage preceding overt morphological divergence, strengthening the developmental interpretation without overextending the scope of the study. Overall, the conclusions are well supported by the data. The manuscript is clearly written, logically organized, and focused. Only minor language refinements are suggested to improve precision and to ensure that claims are carefully aligned with the experimental evidence."

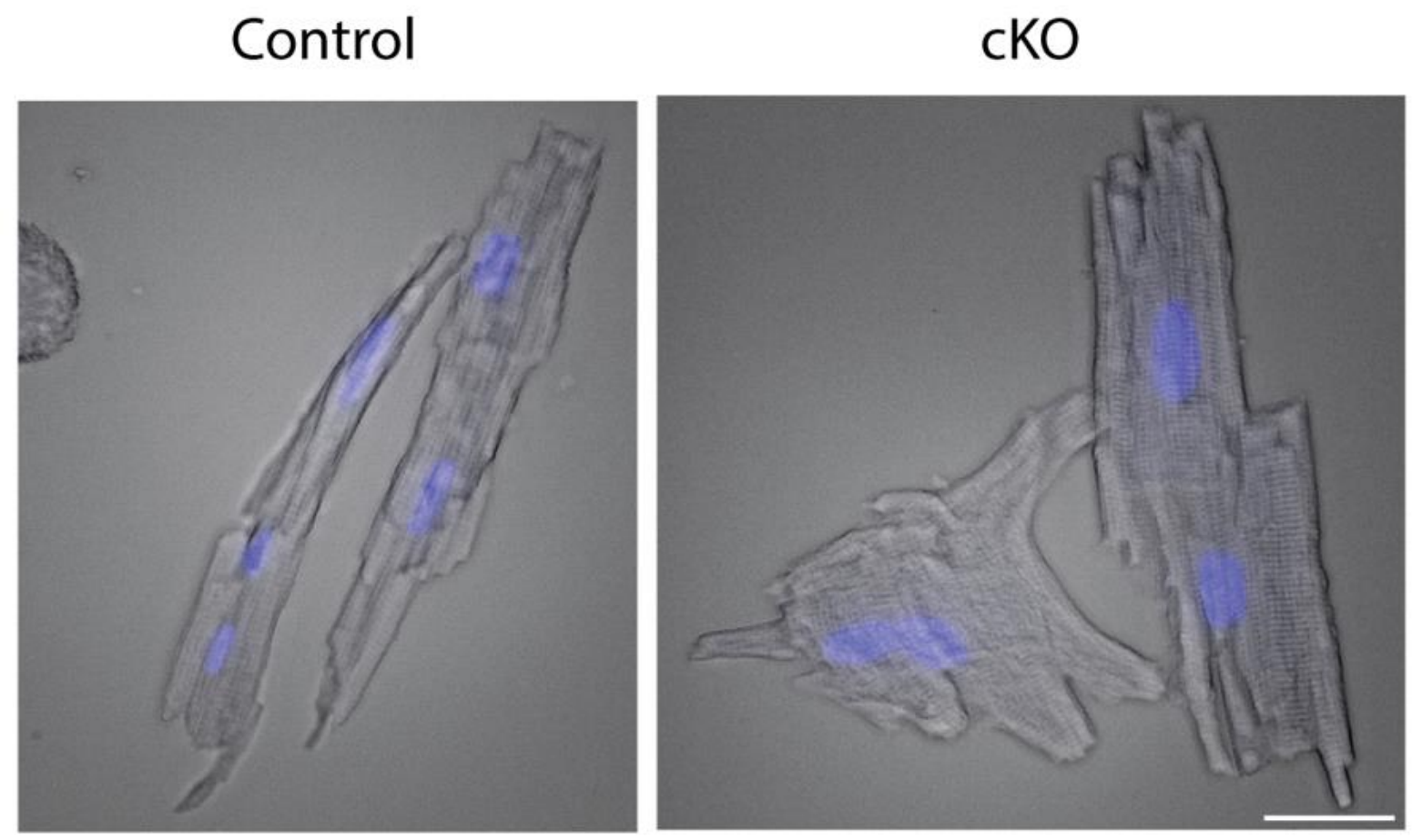

Figure S2. Representative isolated cardiomyocytes used for morphometric analysis. Representative ventricular cardiomyocytes from control and Pkn2 cKO hearts used for the quantitative analysis shown in Figure 1D. Nuclei are indicated (blue). Consistent with the quantitative results, cKO cardiomyocytes appear thicker. Scale bar, 20 μm

Specific comments, with recommendations:

1. Clarify language related to cardiomyocyte migration
Several passages refer to an "early proliferative and migratory phase" or to disruption of "cell migration." While the enrichment of cytoskeletal and motility-associated gene sets supports this interpretation, migration is not directly measured in the study.
Recommendation: Slightly soften these statements by emphasizing cytoskeletal remodeling or motility-associated programs rather than explicit migration. This will improve precision without weakening the central conclusions.

We agree and have softened all language that could be interpreted as directly measuring cardiomyocyte migration. We now emphasize "cytoskeletal remodeling" and "motility-associated programs" instead of explicit migration, consistent with the data presented.

2. Narrow claims regarding dispensability for morphogenesis
Statements indicating that PKN2 becomes dispensable for "heart morphogenesis" or the "remaining morphogenetic program" may read as broader than what was directly assessed.
Recommendation: Specify "gross ventricular morphogenesis" or "ventricular chamber geometry" to align conclusions with the measured outcomes.

We agree and have narrowed the wording throughout to specify that PKN2 becomes dispensable for gross ventricular chamber morphogenesis/geometry (as assessed by our morphometric readouts), rather than for heart morphogenesis broadly.

3. Moderate mechanistic phrasing in the Discussion
The multi-omics analysis provides strong molecular context but does not directly establish downstream mechanisms or effectors.
Recommendation: Replace phrases such as "provides a mechanistic rationale" with "provides molecular context" or "identifies molecular programs associated with the phenotype."

We agree and have moderated mechanistic phrasing in the Discussion. We now describe the multi-omics results as providing molecular context and identifying programs associated with the phenotype, rather than implying definitive downstream mechanisms or effectors.

4. Minor clarity and wording suggestions
Replace "unbiased stage for molecular profiling" with "a stage prior to overt morphological divergence."
Correct minor typographical issues (e.g., missing words related to postnatal timing).
Ensure consistent use of plural forms (e.g., "gene sets") and hyphenation (e.g., "multi-omics," "light-sheet microscopy").

We implemented the suggested wording refinements and corrected minor typographical issues. For example, we replaced "unbiased stage for molecular profiling" with "a stage prior to overt morphological divergence", and ensured consistent terminology and hyphenation (e.g., "multi-omics", "light-sheet microscopy", and "gene sets").

Reviewer #2

We thank the reviewer for recognizing the strengths of the study and for the constructive suggestions, which we address point-by- point below.

Major concerns:

1. Limited mechanistic insight. Cytoskeletal involvement is inferred from enrichment analysis; no direct functional evidence (e.g., actin organization, migration, polarity).

We agree that cytoskeletal involvement is inferred from enrichment analysis. The revised Discussion now explicitly frames our cytoskeletal findings as cross-modality pathway-level signatures rather than direct mechanistic measurements. Direct functional testing of actin organization, polarity, and migratory behavior in PKN2-deficient cardiomyocytes by targeted live-imaging and cell-biological assays is identified as the natural mechanistic follow-up to this work in the new "Limitations and future directions" paragraph of the Discussion.

2. Overinterpretation of multi-omics data. Few strong individual changes at E10.5, yet strong conclusions are drawn from pathway analysis.

We have softened causal phrasing in the Abstract and Introduction to pathway-level language ("transcriptional signatures of…", "consistent with…"). Tracked changes are visible in the revised manuscript.

3. Modest proliferation defect. The ~20% reduction may not fully explain the strong morphological phenotype.

We agree that the proliferation defect alone is unlikely to account for the full morphological phenotype. The revised Discussion now explicitly frames the proliferation reduction as one component of a multifactorial defect that compounds with concurrent cytoskeletal program changes across the narrow morphogenetic window.

4. Temporal window not fully resolved. Only two induction time points (E7.5 vs E10.5) are tested.

The two induction time points (E7.5, E10.5) were chosen to bracket the window identified by our morphological and multi-omics analyses, and they are sufficient to establish that PKN2 dependence is restricted to an early phase. We now state in the Discussion that finer temporal resolution will refine the boundaries of this window in future work.

5. Potential confounders. Genetic background effects and possible non-cell-autonomous contributions are not fully addressed.

The genetic-background point is already addressed in the existing Discussion paragraph contrasting our C57BL/6N line with the Marshall et al. C57BL/6J line and citing known substrain divergence; all comparisons in this study used littermate controls. Regarding cellular specificity, the cardiomyocyte-specific Cre drivers (XMLC2-Cre, Tnnt2-MerCreMer) restrict PKN2 deletion to cardiomyocytes, so the phenotype reflects loss of PKN2 within the cardiomyocyte lineage.

Minor comments:

Clarify statistical methods and sample sizes

Sample sizes, tests, and significance thresholds are reported in each figure legend and detailed in the Statistics section of the Methods; the legend for Figure 1D has been updated to specify the

number of mice and cells per mouse contributing to the morphometric analysis. Software versions and key parameters are specified in Methods.

Improve figure clarity (especially gene set enrichment plots)
Figure 2C has been updated: the legend now defines the "Phospho" abbreviation and clarifies that boxes mark gene sets with directionally concordant enrichment across all three modalities.

Provide more methodological detail for reproducibility
Methods are reported in detail, including software versions, significance thresholds, model specifications, and pipeline parameters for each modality. We have re-reviewed the Methods to confirm completeness.

Reviewer #2

We thank the reviewer for the positive assessment.
